# Aversive Learning and Appetitive Motivation Toggle Feed-Forward Inhibition in the *Drosophila* Mushroom Body

**DOI:** 10.1016/j.neuron.2016.04.034

**Published:** 2016-06-01

**Authors:** Emmanuel Perisse, David Owald, Oliver Barnstedt, Clifford B. Talbot, Wolf Huetteroth, Scott Waddell

**Affiliations:** 1 Centre for Neural Circuits and Behaviour, The University of Oxford, Tinsley Building, Mansfield Road, Oxford, OX1 3SR, UK

## Abstract

In *Drosophila*, negatively reinforcing dopaminergic neurons also provide the inhibitory control of satiety over appetitive memory expression. Here we show that aversive learning causes a persistent depression of the conditioned odor drive to two downstream feed-forward inhibitory GABAergic interneurons of the mushroom body, called MVP2, or mushroom body output neuron (MBON)-γ1pedc>α/β. However, MVP2 neuron output is only essential for expression of short-term aversive memory. Stimulating MVP2 neurons preferentially inhibits the odor-evoked activity of avoidance-directing MBONs and odor-driven avoidance behavior, whereas their inhibition enhances odor avoidance. In contrast, odor-evoked activity of MVP2 neurons is elevated in hungry flies, and their feed-forward inhibition is required for expression of appetitive memory at all times. Moreover, imposing MVP2 activity promotes inappropriate appetitive memory expression in food-satiated flies. Aversive learning and appetitive motivation therefore toggle alternate modes of a common feed-forward inhibitory MVP2 pathway to promote conditioned odor avoidance or approach.

## Highlights

• Aversive learning reduces odor-specific feed-forward inhibition in mushroom body
• Feed-forward inhibition selectively inhibits avoidance-directing neural pathways
• Appetitive motivation increases feed-forward inhibition in the mushroom body
• Imposing feed-forward inhibition favors appetitive memory expression

## Teaser

Fruit fly memory and its state-dependent behavioral expression involve modulation of mushroom body output synapses. Perisse et al. demonstrate aversive learning and appetitive motivation toggle alternate modes of feed-forward inhibition in mushroom body, favoring either conditioned avoidance or approach behavior.

## Introduction

Learning and internal states guide appropriate behavior by altering the properties of neural circuits. A great number of studies across phyla have elucidated brain structures and cellular mechanisms that underlie these changes, but we still know relatively little about how experience and states are implemented in the functional connectivity of a neural network. Inhibition across a range of timescales from milliseconds to days, mediated by neurotransmitters, neuromodulators, and a variety of neuropeptides, is emerging as a critical and general operating principle of neural circuit function and behavioral control (Klausberger and Somogyi, 2008, Fishell and Rudy, 2011, Letzkus et al., 2015).

Fast inhibition can spatially and temporally refine neural representations of sensory stimuli so that specificity is maintained and windows of time in which neural integration can take place are established (Gabernet et al., 2005). In addition, fast and persistent inhibition can alter neural excitability and the efficacy of synaptic transmission and thereby re-route the flow of information through circuits (Vogels and Abbott, 2005, Schwab and Houk, 2015). It is therefore important to understand the mechanisms that control, and the circumstances in which, the level of inhibition is altered in the nervous system.

The reduced numerical complexity of the *Drosophila* brain permits an understanding of these mechanisms at cellular resolution. Studies in flies, mice, and primates have established that dopaminergic neurons (DANs) play a critical role in reinforcement and motivation (Schultz et al., 1997, Wise, 2004, Bromberg-Martin et al., 2010, Berridge, 2012, Waddell, 2013). Across phyla DANs appear to be heterogeneous (Matsumoto and Hikosaka, 2009, Lammel et al., 2011, Lammel et al., 2012, Menegas et al., 2015, Beier et al., 2015, Lerner et al., 2015, Mao and Davis, 2009, Claridge-Chang et al., 2009, Krashes et al., 2009, Aso et al., 2010, Aso et al., 2012, Liu et al., 2012, Burke et al., 2012, Riemensperger et al., 2013), and recordings suggest that some DANs respond to reward-related events and others react to aversive, salient, or surprising cues (Schultz, 2015, Matsumoto and Hikosaka, 2009, Cohen et al., 2012, Horvitz, 2000, Matsumoto and Takada, 2013). Genetic approaches in *Drosophila* and mice revealed that DANs that can provide teaching signals to reinforce either appetitive or aversive memories, project to different locations in the brain (Claridge-Chang et al., 2009, Aso et al., 2010, Aso et al., 2012, Liu et al., 2012, Burke et al., 2012, Zweifel et al., 2011, Darvas et al., 2011, Lammel et al., 2011, Lammel et al., 2012). However, it is currently unclear how the processes of reinforcement relate to those of motivational salience.

Olfactory learning in *Drosophila* could provide an inroad. Flies assign negative and positive values to odors in aversive and reward based paradigms (Tully and Quinn, 1985, Tempel et al., 1983). When subsequently tested for odor preference, they either avoid or approach the conditioned odor. Individual odors are uniquely represented as activity in relatively sparse subpopulations of the ∼2,000 intrinsic Kenyon cells (KCs) per hemisphere of the mushroom body (MB), providing cellular specificity to odor memories (Honegger et al., 2011). During learning, odor-activated KCs receive coincident reinforcing input from combinations of positively or negatively reinforcing DANs (Waddell, 2013). Stimuli such as sweet taste, nutrient value, and water activate distinct populations of rewarding DANs in the protocerebral anterior medial (PAM) cluster, which innervate different zones on the horizontal lobes of the MB (Burke et al., 2012, Liu et al., 2012, Lin et al., 2014, Huetteroth et al., 2015, Yamagata et al., 2015). Reward quality therefore seems to be represented in different DANs, and memories of these rewarding stimuli are presumably formed within the relevant orthogonal zones along the odor-activated KC arbor (Owald and Waddell, 2015). In contrast, aversive DANs innervate the heel, peduncle, and vertical lobes of the MB (Riemensperger et al., 2005, Mao and Davis, 2009, Claridge-Chang et al., 2009, Aso et al., 2010), but electric shock, heat, and bitter taste appear to bottle-neck onto the same MP1, also called PPL1-γ1pedc DANs (Aso et al., 2012, Das et al., 2014, Galili et al., 2014), suggesting that aversive memory lacks quality information and might simply represent the magnitude of aversion. Interestingly, studies suggest that negatively reinforcing MP1 DANs also mediate hunger-dependent motivational control over appetitive memory expression (Krashes et al., 2009, Waddell, 2013). In this context, MP1 DANs appear to be inhibitory since blocking them releases inappropriate memory expression in food-satiated flies (Krashes et al., 2009). Moreover, MP1 DANs are themselves controlled by peptidergic inhibition—dNPF, the fly equivalent of NPY—demonstrating that behavior can be controlled through a hierarchical layering of inhibitory pathways.

Each of the 15 discrete MB zones that is defined by the innervation of a particular type of DAN has a corresponding set of MB output neurons (MBONs) (Aso et al., 2014a), suggesting that DANs specifically modulate the efficacy of the KC-MBON connection within a zone (Owald and Waddell, 2015). Indeed, recent work has revealed a clear model for how DAN reinforcement during learning can shape odor-driven behavior (Owald et al., 2015, Owald and Waddell, 2015). Reward learning engages DANs that modulate and suppress the conditioned odor-drive from KCs to glutamatergic MBONs that intrinsically direct avoidance behavior (Owald et al., 2015). In contrast, aversive learning enhances conditioned odor-drive to these avoidance MBONs (Owald et al., 2015, Bouzaiane et al., 2015) while also inhibiting odor-drive to cholinergic (Séjourné et al., 2011), and perhaps GABAergic, MBONs driving approach. Learning and internal states are therefore likely to tune collections of MBON pathways to skew the overall MBON network toward either directing approach or aversion (Owald and Waddell, 2015).

The presynaptic terminals of MP1/PPL1-γ1pedc DANs are intermingled with the dendrites of MVP2 neurons (also called MBON-γ1pedc>α/β; Aso et al., 2014b), consistent with these DANs modifying the KC-MVP2 junction (Owald and Waddell, 2015). Artificial activation of MP1 paired with odor presentation was recently reported to induce an odor-specific depression at this site (Hige et al., 2015). Here we show that aversive learning causes a persistent and specific reduction in the relative conditioned odor drive to MVP2, yet MVP2 output is only required for the expression of short-term aversive memory. Anatomical and functional connectivity suggests that MVP2 exert asymmetric feed-forward inhibition over MBONs on the horizontal and vertical MB lobes, preferentially promoting approach by inhibiting avoidance directing pathways. Consistent with this model, hunger generally increases MVP2 odor-driven responses, and MVP2-dependent inhibition is required for the expression of appetitive memory at all times in hungry flies. Moreover, activation of MVP2 neurons promotes the expression of appetitive memory in food-satiated flies. Aversive learning and appetitive motivation therefore differentially modulate the odor-drive of the MVP2 neurons, which alters feed-forward inhibition onto other MBON pathways within the neural network of the MB. Reduced feed-forward inhibition is required for conditioned avoidance, whereas increased feed-forward inhibition promotes expression of conditioned approach.

## Results

### GAL4 Control of GABA-ergic MVP2 Neurons

**Figure 1:**
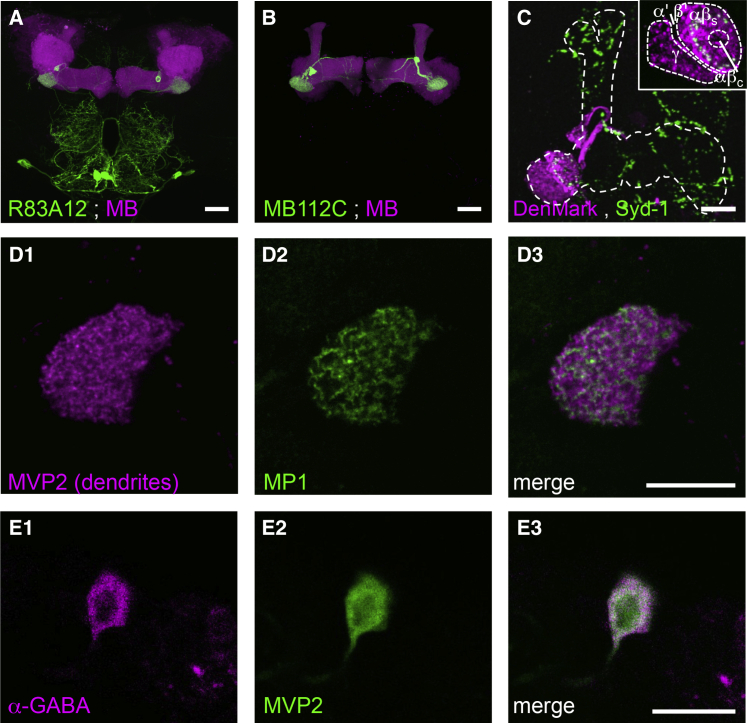
MVP2 MBONs Are Local GABAergic Interneurons of the MB (A and B) (A) R83A12-GAL4- and (B) MB112C-GAL4-driven UAS-mCD8::GFP labels a single MVP2 neuron per hemisphere. The most prominent MVP2 neuron process innervates the heel (γ1) regions of the MB. MB co-labeled (magenta) with 247-LexA::VP16-driven lexAop-rCD2::mRFP. (C) DenMark labels MVP2 dendrites in γ1 and αβ surface at the base of the MB peduncle. The presynaptic active zone marker Syd-1 labels large puncta throughout the α and β lobes and around and outside the MB in the crepine and superior intermediate protocerebrum (see also Figure S1; Movie S1). Inset shows single confocal section through MVP2 dendrites detailing innervation of the γ and αβ_s_, but not αβ_c_ or α′β′ regions. A ring of Syd-1 labeling within the dendritic field suggests MVP2 also feed back within the αβ_s_. Scale bars 20 μm. (D1–D3) (D1) MVP2 dendrites labeled by R12G04-LexA;lexAop-rCD2::mRFP are interspersed with (D2) processes of MP1 DANs labeled by R22B12-GAL4;UAS-mCD8::GFP. (D3) merge of (D1) and (D2); scale bar 10 μm. (E1–E3) (E1) GABA immunostaining overlaps with (E2) MVP2 labeled with MB112C;UAS-mCD8::GFP. (E3) Merge of (E1) and (E2); scale bar 10 μm.

We used the R83A12-GAL4 (Jenett et al., 2012) and the MB112C split-GAL4 combination (Aso et al., 2014b, Aso et al., 2014a) drivers to investigate the role of MVP2 (MBON-γ1pedc>αβ) neurons. Expression of a UAS-CD8::GFP transgene revealed that R83A12 labels the MVP2 neurons in addition to six large cells with processes confined to the sub-esophageal ganglion (Figure 1A). Neural expression in MB112C is restricted to MVP2 neurons (Aso et al., 2014a) (Figure 1B). Double labeling the MB with rCD2::RFP revealed that most MVP2 processes lie within or in close proximity to the structure of the MB lobes, with a few processes projecting outside in the crepine and superior intermediate protocerebrum (Ito et al., 2014, Aso et al., 2014a) (Figures 1A–1C and S1; Movie S1). Expressing the dendritic UAS-DenMark (Nicolaï et al., 2010) and presynaptic UAS-GFP-Syd-1 (Owald et al., 2010) markers in MVP2 neurons with R83A12 control suggests that dendrites of MVP2 occupy the γ1 and base of the peduncle regions of the MB (Figure 1C), where they are interspersed with the processes of the MP1 DANs (Figure 1D), whereas the presynaptic regions are mostly within, or in close proximity to, the MB lobes (Figure 1C). UAS-GFP-Syd-1 also labels a ring of presynaptic active zones at the level of the αβ surface (αβ_s_) neurons, suggesting plausible feedback in this area (inset Figure 1C). GABA immunostaining revealed that MVP2 neurons are likely to be inhibitory (Figure 1E). A prior study concluded that MVP2 neurons predominantly innervate the α1, α2, α3, and β1 and β2 regions of the MB lobes, where they could potentially provide feed-forward inhibition to other MBON compartments (Aso et al., 2014a) (Figure S1).

### Aversive Learning Depresses Conditioned Odor Drive to MVP2 Neurons

**Figure 2:**
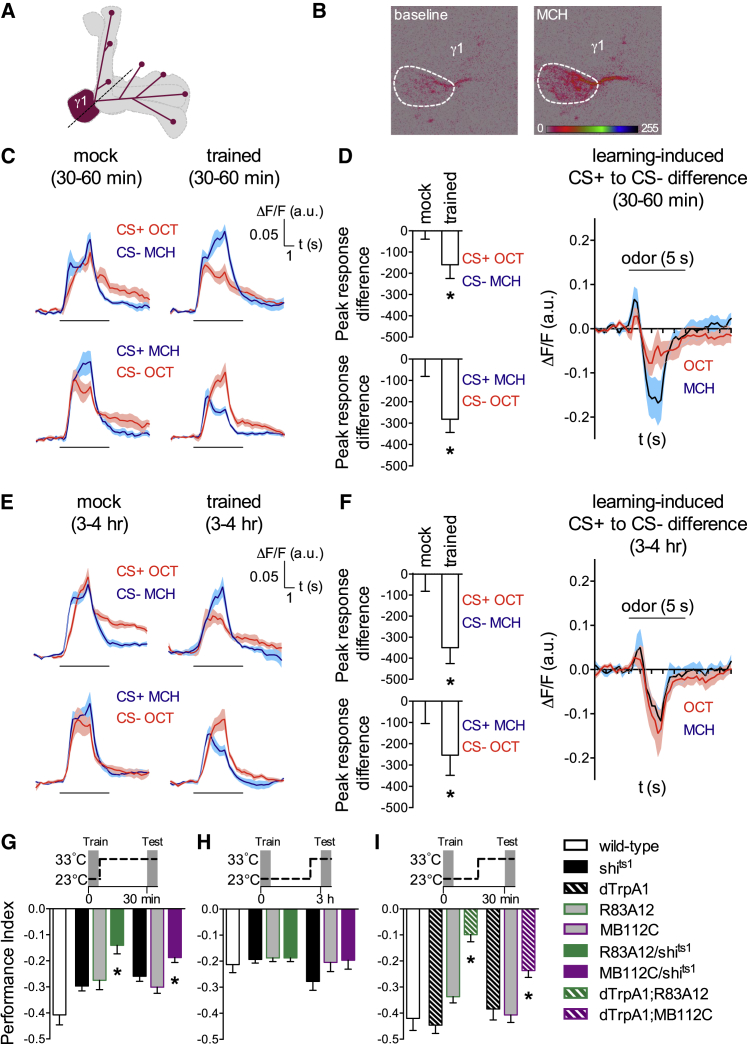
Aversive Learning Drives Persistent Depression of Conditioned Odor Drive to MVP2 Neurons yet Output Is Only Required to Express Short-Term Aversive Memory (A) Schematic of MVP2 neuron showing imaging plane (dotted line). (B) Example pseudocolor images of baseline and MCH-evoked GCaMP fluorescence recorded from MVP2 dendrites in a living fly. ROI indicated by white ellipse. (C) Aversive conditioning depresses the relative CS+ to CS− odor-drive to MVP2 neurons. CS+ and CS− odor-evoked calcium transients were imaged 30–60 min after mock or regular shock conditioning (red curves: OCT, blue curves: MCH). Data are mean [solid line] ± SEM [shaded area] normalized curves (see Experimental Procedures). (D) Bar graphs represent percent difference to the mean mock integrated peak response (4.5 ± 1.5 s after odor delivery, see methods) (Mann-Whitney U-test; OCT is CS+ (top): n(mock) = 7, n(trained) = 11, p < 0.05. MCH is CS+ (bottom): n(mock) = 8, n(trained) = 11, p < 0.05). Difference of responses evoked by CS+ and CS− after aversive conditioning relative to the mean responses after mock training (red curve: OCT is CS+, blue curve: MCH is CS+). Data are mean [solid line] ± SEM [shaded area] normalized curves (see Experimental Procedures). (E and F) Same as in (C) and (D), but odor-responses were imaged 3 to 4 hr after training. Bar graphs represent percent difference to the mean mock integrated peak response (4.5 ± 1.5 s after odor delivery) (Mann-Whitney U-test; OCT is CS+ (top): n(mock) = 5, n(trained) = 5, p < 0.05. MCH is CS+ (bottom): n(mock) = 11, n(trained) = 8, p < 0.05). (G) Blocking output from R83A12 or MB112C neurons during testing impaired 30 min aversive memory performance compared to the relevant controls (Kruskal-Wallis, n = 18–22, p < 0.001 and ANOVA, n = 10–13, p < 0.01, respectively). (H) Blocking output from R83A12 or MB112C neurons during testing did not impair 3 hr aversive memory (Kruskal-Wallis, n = 25, p > 0.9 and ANOVA, n = 9 to 10, p > 0.2, respectively). (I) Activating R83A12 or MB112C neurons during testing impaired 30 min aversive memory (ANOVA, n = 9 to 10, p < 0.001 and ANOVA, n = 13 to 14, p < 0.01, respectively). (G–I) Schematics depict temperature protocols. All flies were trained at 23°C and tested at 33°C. Data are mean ± SEM. See Figure S2 for permissive control. Asterisks indicate statistical significance.

Several reports suggest that learning alters odor-drive to collections of MBONs (Séjourné et al., 2011, Plaçais et al., 2013, Pai et al., 2013, Owald et al., 2015, Bouzaiane et al., 2015) to either skew the overall MB output toward favoring approach or avoidance (Owald et al., 2015, Owald and Waddell, 2015). Since the presynaptic terminals of aversively reinforcing MP1/PPL1-γ1pedc DANs are confined to the same MB zones as the dendrites of MVP2 neurons (Krashes et al., 2009, Aso et al., 2014b) (Figure 1D), we reasoned that aversive learning might alter the KC-MVP2 connection. We therefore measured odor-evoked activity of MVP2 neurons in trained and control flies. We expressed GCaMP6f (Chen et al., 2013) under MB112C control and performed two-photon functional calcium imaging of odor-evoked responses at the level of the MVP2 dendrites in living flies (Figure 2A). We first determined that MVP2 neurons responded to odors, including 4-methylcyclohexanol (MCH) and 3-octanol (OCT) that are typically used for olfactory learning (Figure 2B; also Figure 6A). To test the effect of aversive training, flies were loaded into the training arm of a T-maze and subjected to either of two protocols: the “trained” group received 1 min OCT (or MCH) presentation paired with 12 electric shocks (CS+) followed by 1 min of MCH (or OCT) without reinforcement (CS−); the control “mock” group experienced the same odor regimen but without shock presentation. Flies were subsequently captured and individually mounted under the microscope within 30–60 min after training. Aversive conditioning decreased the response to the CS+ relative to the CS− for the trained groups (Figures 2C and 2D). Importantly, no change was apparent in the responses of mock-trained flies (Figure 2C). As in a previous study (Owald et al., 2015), we also analyzed the difference between the OCT to MCH (or MCH to OCT) responses per individual fly and then compared the averaged difference curves between the trained and the mock-trained groups. Again, a robust depression of the CS+ relative to the CS− was observed for the peak responses of the trained groups (Figure 2D). The observed depression persisted for at least 3 to 4 hr after training (Figures 2E and 2F). These data are consistent with a model that learning drives synaptic weight changes of KC-MBON connections (Okada et al., 2007, Cassenaer and Laurent, 2012, Séjourné et al., 2011, Owald et al., 2015) and with a recent study that reported odor-specific depression following the pairing of odor exposure with artificial stimulation of MP1 DANs (Hige et al., 2015).

### MVP2 Neurons Are Required for Expression of Short-Term Aversive Memory

MP1 DANs mostly reinforce short-term aversive memory (Aso et al., 2012). We therefore tested the requirement of MVP2 neurons in aversive memory by blocking their output during memory testing using R83A12 and MB112C to express the dominant temperature-sensitive UAS-*shibire*^ts1^ (*shi*^ts1^) (Kitamoto, 2001). In each experiment we compared the performance of flies with MVP2 neural blockade to controls carrying only the GAL4 or UAS-*shi*^ts1^ transgene. We first tested 30 min aversive memory performance by training flies at permissive 23°C and raising them to restrictive 33°C before and during memory testing (Figure 2G). Performance of R83A12;*shi*^ts1^ and MB112C;*shi*^ts1^ flies with impaired MVP2 neurons was statistically different to that of their respective controls. Importantly, experiments performed at permissive 23°C throughout did not reveal significant differences between the relevant groups (Figure S2A). We next tested the requirement of MVP2 neurons for 3 hr aversive memory. Flies were trained at permissive 23°C and raised to restrictive 33°C 30 min before and during testing. Strikingly, performance of R83A12;*shi*^ts1^ flies with blocked MVP2 neurons was statistically indistinguishable from that of control flies at this time (Figure 2H). Therefore, although the decrease in conditioned odor-drive to MVP2 neurons persists, MVP2 output is only essential for the expression of short-term aversive memory. These data are consistent with MP1 DANs principally reinforcing short-term memory by modifying the KC-MVP2 junction, whereas expression of later phases of aversive memory relies on other pathways such as V2α MBONs on the vertical MB lobes (Séjourné et al., 2011, Bouzaiane et al., 2015).

We also tested whether MVP2 neuron stimulation with UAS-*dTrpA1* (Hamada et al., 2008) altered expression of aversive memory. Flies in which MVP2 neurons were activated 15 min prior to and during testing aversive memory showed a significant decrease in performance compared to controls (Figure 2I). Importantly, no significant differences were apparent if the experiment was performed at 23°C throughout (Figure S2B). We note that stimulating MVP2 neurons during memory testing produced a similar defect to that obtained when MVP2 neurons were blocked. A plausible explanation is that, when blocked, the flies cannot transmit the learned relative odor-specific drive from KCs to MVP2 neurons to the relevant downstream neurons. Similarly, when the MVP2 neurons are continuously stimulated the relative odor-specificity of MVP2 activity is lost.

### MVP2 Neurons Asymmetrically Inhibit the MBON Network

**Figure 3:**
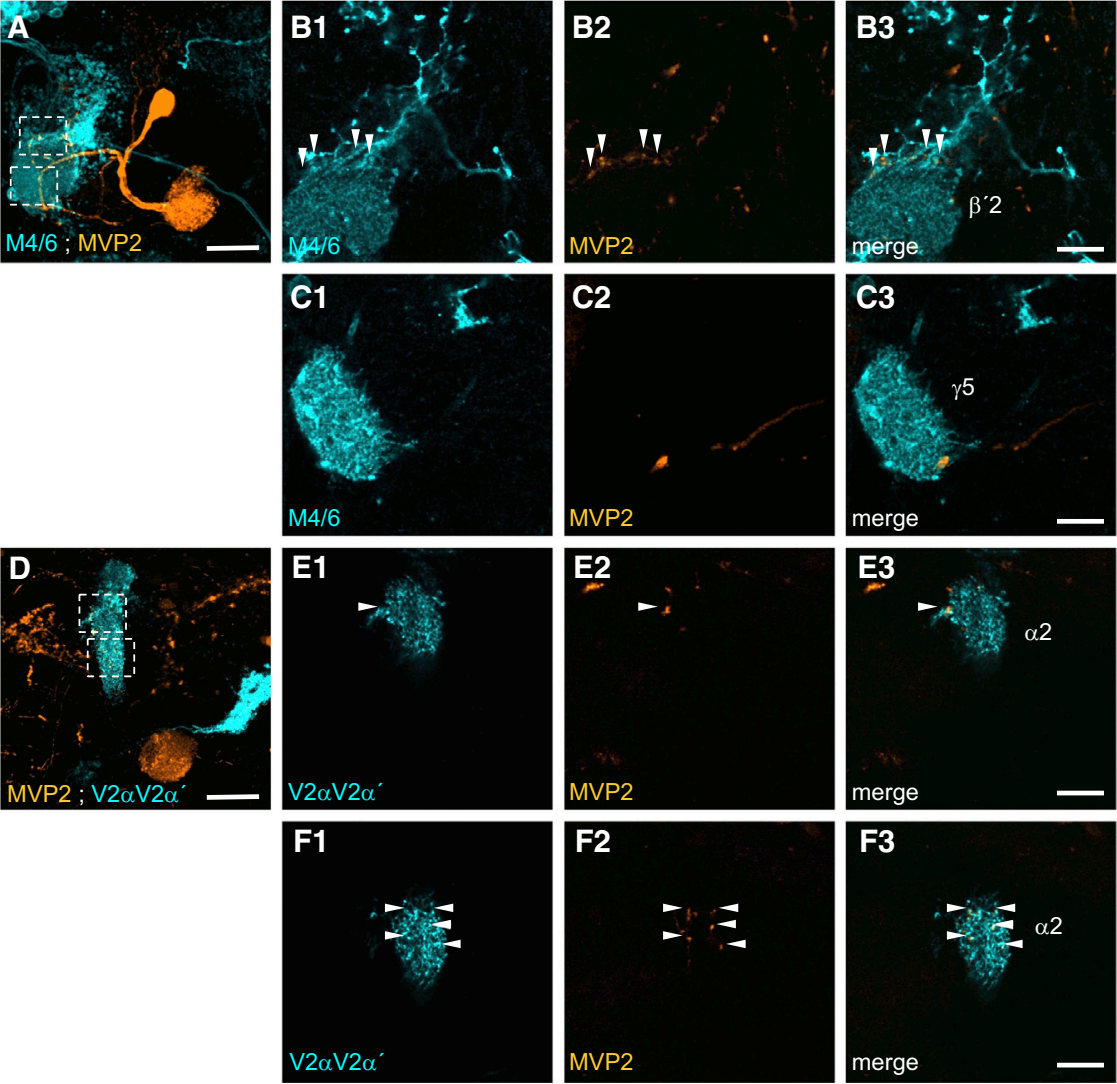
Anatomy of MVP2 Processes in Relation to M4/6 and V2αV2α′ MBONs (A) Confocal projection of single MVP2 neuron with the M4/6 neurons labeled with R83A12-GAL4; UAS-GCaMP6f (orange) and R21D02-LexA; lexAop-rCD2::mRFP (cyan), respectively. Scale bar 20 μm. (B1–B3) Separate and merged channels of single confocal section at the level of the M4/6 dendrites in the β′2 zone showing MVP2 processes intermingled with the M4/6 axonal segment (white arrows). Scale bar 10 μm. (C1–C3) Separate and merged channels of single confocal section at the level of the M6 dendrites in the γ5 zone show no overlap with MVP2 processes except for a large diameter neurite passing through. Scale bar 10 μm. Also see Movie S2. (D) Confocal projection of single MVP2 neuron with the V2αV2α′ neurons labeled with R12G04-LexA; lexAop-rCD2::mRFP (orange) and R71D08-GAL4; UAS-mCD8::GFP (cyan), respectively. Scale bar 20 μm. (E1–E3) Separate and merged channels of single confocal section at the level of the V2αV2α′ dendrites in the α2α′2 zone of the vertical MB lobe showing a single MVP2 process close to the axonal segment of V2αV2α′ (white arrow). (F1–F3) Separate and merged channels of another single confocal section showing several MVP2 processes (white arrows) within the mass of the V2αV2α′ dendrites. Scale bar 10 μm. Also see Movie S3.

Optogenetic activation of MVP2 drives approach behavior (Aso et al., 2014b), and most MVP2 processes lie within, or in close proximity to the MB. We therefore hypothesized that MVP2 neurons might skew the MBON network toward approach by preferentially inhibiting avoidance-directing MBONs. We first investigated this model by looking at the anatomy of MVP2 presynaptic neurites and MBON processes in the vertical and horizontal MB lobes. We used compatible GAL4 and LexA drivers to co-label MVP2 neurons with either the M4/6 MBONs on the horizontal lobes or the V2α and V2α′ MBONs on the vertical lobes (Figure 3). These confocal analyses suggest that MVP2 presynaptic terminals lie mostly outside the main dendritic fields of the M4/6 neurons in the horizontal lobe tips and instead appear clustered on the M4/6 neurites as they exit the MB lobe region (Figures 3A–3C; Movie S2). In contrast, many MVP2 terminals lie within the MB neuropil occupied by dendrites of V2α and V2α′ MBONs (Figures 3D–3F; Movie S3).

**Figure 4:**
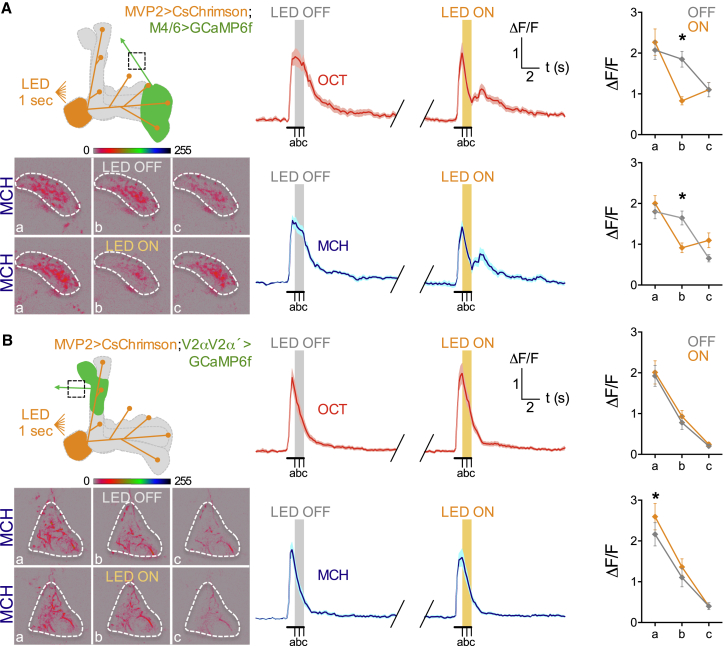
MVP2 Neurons Inhibit Odor-Evoked Responses in M4/6, but Not V2αV2α′ MBONs Odor-evoked GCaMP6f responses measured in (A) M4/6 or (B) V2αV2α′ MBON axons (green) while CsChrimson-expressing MVP2 MBONs (orange) were light-triggered. (A) Schematic of experiment (top left). Data acquired from the most ventral part of M4β′ axons (dashed square). Lower left panels; representative images taken at time points a, b, and c, after MCH presentation without and with stimulation of MVP2 neurons. Calcium traces during OCT (middle top panels, red) or MCH (middle bottom panels, blue) presentation show robust odor-evoked responses in M4β′ axons in absence of LED stimulation. Triggering MVP2 neurons for 1 s with LED ON produced a clear and reversible depression of the odor-evoked calcium transient. Data are mean curves [solid line] ± SEM [shaded area]. Quantification of the ΔF/F at the a–c time points reveals a significant difference in the odor-evoked responses with LED ON (orange) compared to the same time point with LED OFF (gray), for both OCT (top right) and MCH (bottom right) (two-way repeated-measures ANOVA, both interaction effect p < 0.001, n = 9). (B) Schematic of experiment (top left). Data were acquired from the V2αV2α′ proximal axon segment (dashed square). Bottom left panels; representative images taken at time points a, b, and c, after MCH presentation without and with stimulation of MVP2 neurons. Calcium traces during OCT (middle top panels, red) or MCH (middle bottom panels, blue) presentation show robust odor-evoked responses in V2αV2α′ axons without and with LED-triggered stimulation of MVP2 neurons. Data are mean curves [solid line] ± SEM [shaded area]. Quantification of the ΔF/F at the a–c time points reveals no significant difference in the odor-evoked responses with LED ON (orange) compared to the same time point with LED OFF (gray), for both OCT (top right) and MCH (bottom right) (OCT: two-way repeated-measures ANOVA, no interaction effect p > 0.6, n = 13; MCH: Two-way repeated-measures ANOVA, interaction effect p < 0.05, “a” LED OFF versus LED ON, p = 0.001, n = 13).

Since the detail of light microscope level anatomy is limited, we next used odor-evoked activity and optogenetic control of MVP2 neurons to test for functional connectivity between MVP2 and M4/6 or V2αV2α′ MBONs (Figure 4). Flies were constructed that expressed GCaMP6f in M4/6 or V2αV2α′ MBONs using either R21D02-GAL4 or R71D08-GAL4, respectively, and CsChrimson (Klapoetke et al., 2014, Hoopfer et al., 2015) in MVP2 neurons under R12G04-LexA. We then monitored MCH- or OCT-evoked responses in the presynaptic processes of M4/6 or V2αV2α′ MBONs before, during and following red-light-triggered MVP2 activation. Strikingly, whereas MVP2 activation induced a rapid and robust inhibition of OCT- and MCH-evoked responses in M4/6 MBONs (Figure 4A) that recovered after the activation ended, no effect was evident in V2αV2α′ responses (Figure 4B). Importantly, flies lacking retinal or the CsChrimson transgene did not exhibit a measurable difference on OCT- or MCH-evoked responses in M4/6 MBONs (Figures S3A and S3B). Moreover, stimulating MVP2 neurons without concurrent odor delivery did not induce a measurable M4/6 calcium response (Figure S3C). These data are consistent with MVP2 neurons preferentially inhibiting horizontal lobe MBONs. In addition, since aversive learning reduces conditioned odor-drive to MVP2 neurons, disinhibition might explain why aversive learning caused a relative increase in conditioned odor-evoked responses in M4/6 neurons (Owald et al., 2015).

**Figure 5:**
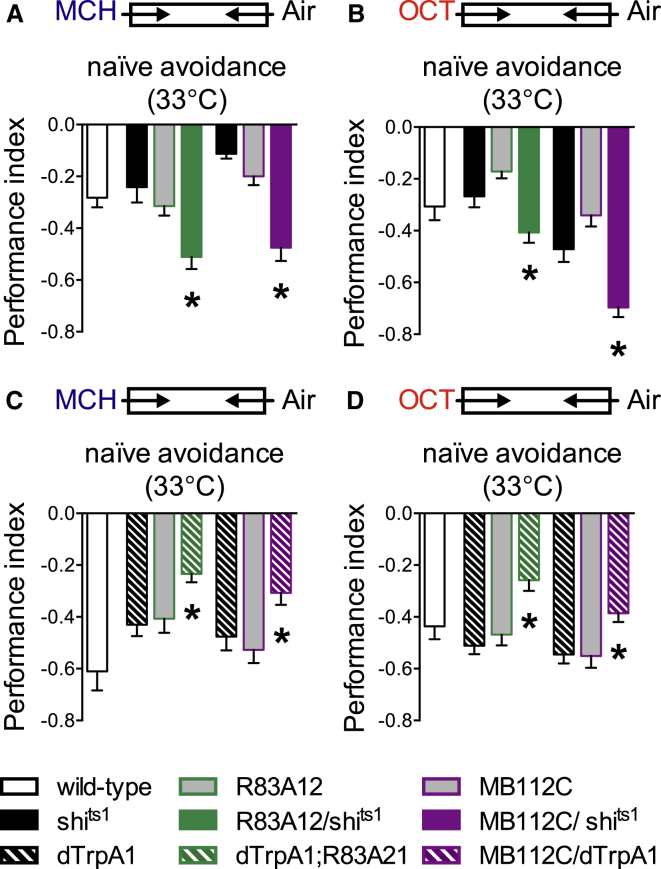
MVP2 Neurons Inhibit Naive Odor Avoidance Behavior (A and B) Blocking MVP2 neuron output in naive flies increases odor avoidance for (A) MCH (R83A12: ANOVA, n = 10–12, p < 0.01; MB112C: ANOVA, n = 8–12, p < 0.01) and for (B) OCT (R83A12: ANOVA, n ≥ 16–20, p < 0.01; MB112C: ANOVA, n = 15 to 16, p < 0.01). (C and D) Stimulating MVP2 neurons in naive flies inhibits odor avoidance for (C) MCH (R83A12: Kruskal-Wallis, n = 10–12, p = 0.01; MB112C: ANOVA, n = 8–12, p < 0.01) and for (D) OCT (R83A12: ANOVA, n = 14–16, p < 0.01; MB112C: Kruskal-Wallis, n = 16–20, p < 0.01). Flies chose between T-maze arms containing MCH or OCT or a clean air stream at 33°C. See Figure S4 for permissive controls.

Naive odor-driven behavior can be steered by skewing the balance of the outputs in the overall MBON network (Owald and Waddell, 2015). Blocking either input or synaptic output from the M4/6 MBONs converts naive odor avoidance into approach (Barnstedt et al., 2016, Owald et al., 2015). We therefore also used naive odor-avoidance behavior to test whether MVP2 neurons exert asymmetric influence on the MBON network. We expressed UAS-*shi*^ts1^ with R83A12 or MB112C and determined the effect on naive odor avoidance of blocking MVP2 neurons (Figure 5). Flies chose between T-maze arms containing clean air or with MCH or OCT. Both R83A12;*shi*^ts1^ and MB112C;*shi*^ts1^ flies exhibited significantly enhanced MCH (Figure 5A) and OCT (Figure 5B) avoidance behavior at restrictive 33°C but not permissive 23°C (Figures S4A and S4B). We also expressed *dTrpA1* in MVP2 neurons and tested whether stimulating MVP2 neurons suppressed naive odor avoidance behavior. Whereas all flies robustly avoided MCH or OCT at 23°C (Figures S4C and S4D), at restrictive 33°C, R83A12;*dTrpA1* and MB112C;*dTrpA1* flies displayed significantly weaker avoidance of MCH (Figure 5C) and OCT (Figure 5D). Taken with the aversive memory defect seen when MVP2 neurons are blocked (Figure 2G) and the structural and functional anatomy (Figures 3 and 4), these naive fly data are consistent with GABA-ergic MVP2 neurons skewing the MBON network by preferentially inhibiting MBON pathways that generate avoidance behavior.

### Hunger Potentiates Odor-Evoked Activity of MVP2 Neurons

**Figure 6:**
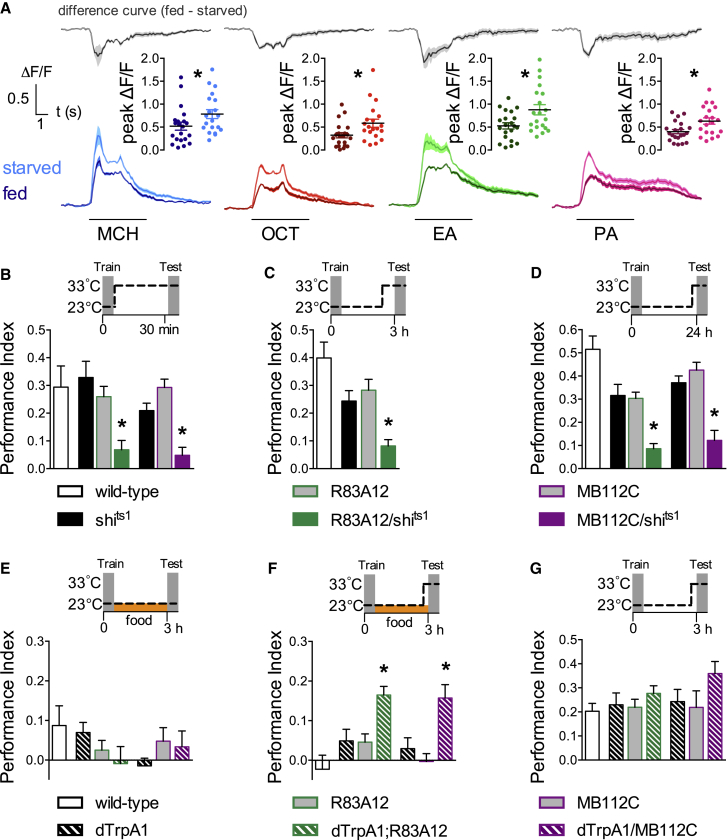
Elevated MVP2 Activity Promotes Appetitive Memory Expression in Hungry Flies (A) Hunger increases odor-evoked responses in MVP2 neurons. Peak responses in starved flies (light curves; mean [solid line] ± SEM [shaded area]) are statistically different to those in fed flies (dark curves; mean [solid line] ± SEM [shaded area]). Flies were exposed to 4-methylcyclohexanol (MCH), 3-octanol (OCT), ethylacetate (EA), or pentylacetate (PA) for 5 s. Top: difference curves (gray) between the mean responses from fed and starved flies. Insets show quantification of peak responses (4.5 ± 1.5 s after odor delivery, see Experimental Procedures; asterisks denote statistical significance; Mann-Whitney U-tests; all n(starved) = 20, n(fed) = 21, p < 0.05). (B–D) Blocking MVP2 output during memory testing impairs appetitive memory performance at all times. Flies were trained at 23°C and raised to 33°C before and during testing (B) 30 min, (C) 3 hr, or (D) 24 hr memory. Performance of MVP2;UAS-*shi*^ts1^ flies was statistically different from controls for (B) (R83A12: Kruskal-Wallis, n = 9, p < 0.01. MB112C: Kruskal-Wallis, n = 8 to 9, p < 0.01), (C) (R83A12: ANOVA, n ≥ 11, p < 0.01), and (D) (R83A12: ANOVA, n = 10–12, p < 0.01. MB112C: ANOVA, n = 10–12, p < 0.01). (E) Feeding flies after training suppresses appetitive memory performance. Hungry flies were trained at 23°C, then stored in food vials before testing 3 hr memory at 23°C. No statistical differences were apparent between flies expressing UAS-*dTrpA1* in MVP2 neurons and relevant controls (R83A12: ANOVA, n = 10, p > 0.2; MB112C: ANOVA, n = 9 to 10, p > 0.3). (F) Appetitive memory expression is promoted in fed flies by activation of MVP2 neurons 15 min prior to and during testing 3 hr memory (R83A12: ANOVA, n = 11–19, p < 0.01; MB112C: ANOVA, n = 14–17, p < 0.01). Hungry flies were trained, then stored in food vials before testing 3 hr memory at 33°C. (G) Activating MVP2 neurons does not further enhance appetitive memory performance in hungry flies (R83A12: ANOVA, n = 9 to 10, p > 0.5; MB112C: Kruskal-Wallis, n = 8–10, p > 0.1). Hungry flies were trained, stored in empty vials and tested for 3 hr memory at 33°C. All data are mean ± SEM. Schematics illustrate the temperature protocols. See Figure S5 for permissive temperature controls.

In addition to conveying negative reinforcement, the MP1 DANs inhibit expression of sugar-reinforced appetitive memory in food-satiated flies (Krashes et al., 2009). Furthermore, MP1 DANs are more active in food-satiated than in hungry flies (Plaçais and Preat, 2013). We therefore tested whether hunger modulated MVP2 activity by monitoring odor-evoked responses in hungry and food-satiated flies (Figure 6A). We again expressed GCaMP6f in MVP2 neurons using MB112C. Flies were either housed in food vials and allowed to feed ad libitum (fed) or were stored in vials with 1% agar as a water source and deprived of food for 22–26 hr (starved) before being prepared for live imaging. These experiments revealed a clear elevation of odor-evoked activity in starved compared to satiated flies. Peak responses to MCH, OCT, ethyl acetate (EA), and pentyl acetate (PA) were all significantly greater in starved versus satiated flies. 6-methyl-5-heptan-2-one and geranylacetate showed a trend toward increased responses in starved flies but did not reach statistical significance (data not shown). The shape of the responses, an odor-specific signature, appeared to be preserved in fed and starved flies. These data suggest that hunger increases general odor-drive from KCs to MVP2 neurons—an expectation of a release of MP1-directed modulation of the KC-MVP2 junction—and thereby increases feed-forward inhibition in the MBON network.

We also tested whether appetitive conditioning altered relative odor-drive to MVP2 neurons (Figures S5A and S5B). Flies were again subjected to either of two protocols: the “trained” group received 2 min OCT (or MCH) without reinforcement (CS−) followed by 2 min of MCH (or OCT) paired with sucrose (CS+); the “mock” group experienced the same odor regimen but without reinforcer. Flies were individually mounted under the microscope 30–60 min after training. The averaged difference curves between the trained and the mock-trained groups revealed a potentiation of the CS+ relative to the CS− for the peak responses of the OCT but not the MCH-trained groups (Figures S5A and S5B). We conclude that appetitive conditioning may potentiate the relative conditioned odor-drive to MVP2 neurons.

### MVP2 Neurons Are Generally Required for the Expression of Appetitive Memory

Since MVP2 neurons are more excitable in hungry flies, we reasoned that their output might be required to promote state-dependent appetitive memory expression by inhibiting avoidance-directing MBONs. We therefore used R83A12-and MB112C-driven UAS-*shi*^ts1^ to assess the role of MVP2 neurons in appetitive memory. All flies were food-deprived and trained with odor and sugar at permissive 23°C, after which they were raised to restrictive 33°C 30 min before and during testing 30 min, 3 hr, or 24 hr appetitive memory. Performance of flies with blocked MVP2 neurons was statistically different to that of their respective controls at every time point (Figures 6B–6D). Experiments performed at 23°C throughout did not reveal significant differences between the relevant groups (Figures S5C–S5E). Therefore, whereas MVP2 neurons only contribute to the expression of short-term aversive memory, they are required for flies to express all phases of sugar-reinforced appetitive memory.

### MVP2 Activation Promotes Appetitive Memory Expression in Food-Satiated Flies

We hypothesized that blocking MVP2 output might impair appetitive memory performance because the flies are effectively stuck in a food-satiated condition. To test this idea, we used R83A12- and MB112C-driven expression of *dTrpA1* to activate MVP2 in food-satiated flies before and during assaying memory performance. These flies and all controls were food-deprived and trained with odor and sugar at 23°C. After training, flies were transferred to 23°C food vials and were either kept at this condition before testing 3 hr memory (Figure 6E) or were raised to 33°C 15 min before and during testing 3 hr memory (Figure 6F). Feeding after training suppressed performance in all groups except the R83A12;*dTrpA1* and MB112C;*dTrpA1* flies that were exposed to elevated temperature prior to and during testing (Figures 6E and 6F). Therefore, MVP2 neuron activation promotes inappropriate appetitive memory expression in food-satiated flies. We also used R83A12;*dTrpA1* and MB112C;*dTrpA1* to stimulate MVP2 neurons prior to 3 hr memory testing in food-deprived flies (Figure 6G). No significant improvement in memory performance was apparent when MVP2 neurons were stimulated in this condition.

In parallel, we reproduced the finding that MP1 DAN block promotes appetitive memory performance in satiated flies (Krashes et al., 2009). We used the same food deprivation and training conditions as for the above MVP2 experiments, but expressed UAS-*shi*^ts1^ in MP1 DANs using c061-GAL4;MBGAL80 (Figures S5F–S5H). In these experiments, only satiated c061;MBGAL80; *shi*^ts1^ flies that were exposed to elevated temperature 30 min prior to and during testing displayed robust appetitive memory performance (Figures S5F and S5G). As for MVP2 neuron activation, blocking MP1 neurons in hungry flies did not further enhance appetitive memory performance (Figure S5H). Therefore, MP1 inhibition and MVP2 activation promote appetitive memory performance, consistent with the MP1:KC:MVP2 pathway representing a key part of the state of hunger in the neural circuitry of the MB.

## Discussion

Prior work in *Drosophila* indicated that negative reinforcement and hunger-state-dependent motivational control of appetitive memory performance might be controlled by the same DANs (Krashes et al., 2009, Claridge-Chang et al., 2009, Aso et al., 2010, Aso et al., 2012). The presynaptic field of the MP1/PPL1-γ1pedc DANs occupies a defined region of the MB that also contains the MVP2/MBON-γ1pedc>αβ dendrites (Krashes et al., 2009, Aso et al., 2010, Aso et al., 2014b), suggesting that these DANs modulate the efficacy of this specific KC-MBON connection. Our results here demonstrate that the MVP2 MBONs also play a critical role in the expression of short-term aversive memory and the state-dependence of appetitive memory expression. Since these findings directly mirror the described roles for the MP1 DANs (Krashes et al., 2009), we conclude that DAN modulation of the KC-MVP2 junction is critical for both negative reinforcement during olfactory learning and the motivational salience of appetitive odor cues.

The GABA-ergic MVP2 neurons have postsynaptic and presynaptic processes in the MB, suggesting that they are interneurons of the MB and feed-forward inhibit other MBON compartments. Dendrites of MVP2 neurons (and the presynaptic terminals of the MP1 DANs) innervate the γ1 region and more densely innervate the αβ_s_ than the αβ core (αβ_c_) region of the αβ ensemble (Krashes et al., 2009). MVP2 are therefore likely to be primarily driven by αβ_s_ KCs. Since αβ_s_ neurons contribute to conditioned approach and avoidance, whereas αβ_c_ are particularly important for conditioned approach (Perisse et al., 2013), there is an imbalance in the drive to approach and avoidance behaviors at this level of the MBON network.

Artificial activation of MVP2 neurons in naive flies drives approach behavior (Aso et al., 2014b), consistent with them preferentially inhibiting MBON compartments that direct avoidance—as opposed to those that drive approach. Our anatomical and functional connectivity and odor-directed behavioral data are consistent with such a model. MVP2 stimulation inhibits odor-evoked activity in M4/6 but not in V2αV2α′ MBONs. MVP2 stimulation also promotes expression of approach memory in food-satiated flies, yet it inhibits naive odor avoidance behavior. We conclude that MVP2 directly inhibit the M4/6 class of horizontal lobe MBONs through synapses made on the primary axonal segment as it exits the MB lobes. Inhibition exerted in this area might be expected to control the gain of the MBON responses following integration of KC inputs in the MBON dendrite in a manner similar to perisomatic inhibition in mammals (Miles et al., 1996, Klausberger and Somogyi, 2008, Ellender and Paulsen, 2010). Consistent with this anatomy and idea, we and others (Lewis et al., 2015) did not find obvious changes in the odor drive to the dendritic region of M4/6 neurons between hungry and satiated flies (data not shown), but a hunger-dependent decrease was apparent when odor-evoked responses were measured in the efferent neurites (Figure S6A). In contrast, MVP2 neurons do not functionally inhibit or densely innervate the neurites of V2αV2α′ MBONs, nor does hunger reduce odor-evoked responses in V2αV2α′ MBONs (Figure S6B). It therefore seems likely that MVP2 neurons contact DANs or other neurons that occupy the α2 compartment of the MB lobe (Aso et al., 2014a).

**Figure 7:**
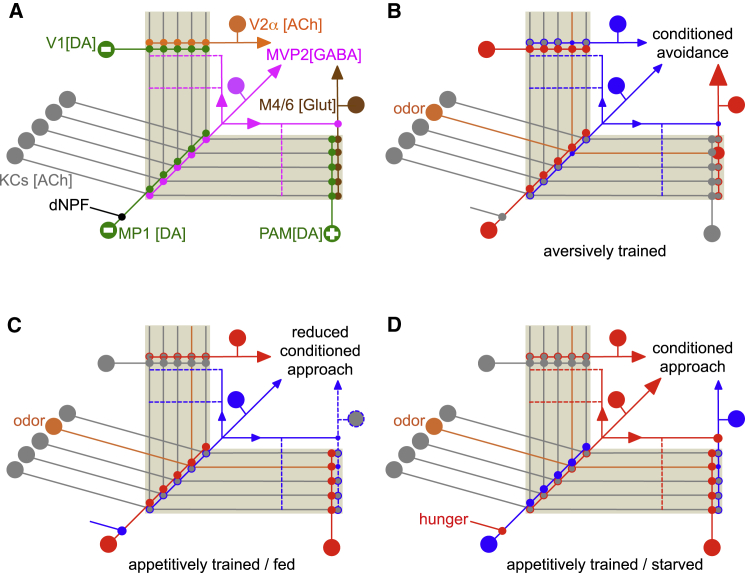
Model Accommodating Role for MVP2 in Aversive Learning and Appetitive Motivation (A) Wiring diagram of the relevant neurons in the MB network. The MP1/PPL1-γ1pedc DANs (green) have a dual role in aversive reinforcement and appetitive motivation and are modulated by the hunger-sensitive dNPF-releasing neurons (black). MP1 and V1 DANs convey negative reinforcing properties of aversive stimuli (minus symbol) to specific zones in the MB ensemble where they modulate the connection between odor-activated KCs (gray, inactive) and the GABAergic MVP2/MBON-γ1pedc>α/β interneuron (magenta) or the V2α (orange) MBONs, respectively. Active MVP2 neurons feed-forward inhibit other parts of the MB network where they modulate the odor-drive to some MBONs including the glutamatergic (Glut) M4/6 MBONs (brown) that promote avoidance behavior. It is currently unclear what other MVP2 projections in the horizontal and vertical lobes connect to (dashed lines). Rewarding stimuli activate positively reinforcing DANs of the PAM cluster (green with plus symbol), which modulate connections between KCs and the M4/6 MBONs. We propose that MVP2 neurons exert their function by bridging between MBON compartments that each have their own DAN input. (B) Mode of the network evoked by aversive training. Red symbolizes high and blue low neural activity. Size of arrowhead indicates relative drive. Sites of plasticity denoted by a change in the size of synaptic connections (circles), with smaller representing depression and larger representing potentiation. For simplicity, only an effect on the neurons carrying the conditioned odor is illustrated. During aversive conditioning, coincidence between odor-driven activity in KCs (now orange) with phasic MP1 and MV1-released dopamine leads to odor-specific synaptic depression (smaller blue circles) between odor-activated KC and the MVP2 and V2α MBONs. After training, the reduced conditioned-odor drive to MVP2 (now blue) selectively weakens feed-forward inhibition onto the conditioned-odor drive to M4/6 MBONs (red) that favor avoidance behavior. Via this feed-forward inhibitory mechanism, MP1-induced synaptic depression at the KC-MVP2 junction is sign-inverted to an apparent potentiation of the KC-M4/6 junction (larger red circle)—importantly, while odor-specificity is maintained. (C) Mode of the network following appetitive training and feeding. During appetitive conditioning coincidence between odor-driven activity in KCs with phasic PAM-released dopamine leads to odor-specific synaptic depression (smaller blue circle) between odor-activated KC and the M4/6 MBONs. Satiation imposes tonic activity of MP1 (red), which reduces general odor-drive to the KC-MVP2 junction, thereby switching MVP2 into a low mode (now blue). Learning-triggered synaptic depression is effectively neutralized by reduced MVP2-mediated inhibition so that the drive to avoidance MBONs is maintained (now dashed blue arrow) and approach behavior cannot be efficiently expressed. (D) Mode of the network following appetitive training and food deprivation. Hunger triggers dNPF neuron activity (now red) that suppresses MP1 activity (now blue). This leads to general elevation of odor-evoked MVP2 activity (now red), which feeds forward to inhibit the avoidance MBON pathways. Increased feed-forward inhibition, combined with odor-specific synaptic depression at the KC M4/6 junction (small blue circle) further reduces drive of avoidance MBONs and skews the MBON network toward promoting conditioned approach behavior.

Our data also demonstrate that aversive learning reduces the relative conditioned odor drive to MVP2 neurons, which would presumably decrease feed-forward inhibition onto the relevant MBON compartments and thereby render them more responsive to odors. Output from the glutamatergic M4/6 neurons, which are postsynaptic to the KCs in the horizontal tip regions, is required for expression of aversive and appetitive memory. Furthermore, the relative odor-drive to M4/6 neurons was shown to be depressed by reward learning (Owald et al., 2015) and potentiated by aversive learning (Owald et al., 2015, Bouzaiane et al., 2015). Since aversive learning reduces the conditioned odor drive of the MVP2 neuron, we propose that the observed increase in odor-drive to M4/6 after aversive learning results from reduced feed-forward inhibition from MVP2. This would mean that bi-directional output plasticity could emerge via a direct junctional plasticity following reward conditioning, but a network property of reduced MVP2 feed-forward inhibition after aversive conditioning (Figure 7). Such a layered feed-forward network architecture linking one site of DAN-driven KC-MBON plasticity to another KC-MBON connection would provide a means to achieve odor-specific bi-directional plasticity at a particular synaptic junction using dopamine-driven synaptic depression in two different places. We propose that this circuit design principle in which plasticity at one site of a neuron can, via feed-forward inhibition, indirectly alter the efficacy of output elsewhere in the same neuron, could be a general feature in the brain of the fly and other animals. It is possible that the KC-MVP2 junction also exhibits bi-directional plasticity, notably with inverted polarity relative to M4/6 plasticity traces.

The layered network architecture places the aversive memory relevant MVP2 plasticity on top of the M4/6 plasticity that is relevant for appetitive memory (Owald et al., 2015). This organization could accommodate the co-existence of aversive and appetitive olfactory memories following conditioning reinforced by sugar laced with bitter taste (Das et al., 2014). Immediately after such training flies avoid the conditioned odor because the aversive taste memory relieves feed-forward inhibition onto the sites that are depressed by appetitive sugar plasticity and therefore over-rides the expression of approach memory. However, as the aversive memory decays, feed-forward inhibition returns and appetitive memory is then expressed. A similar mechanism might account for the time-dependent switch from conditioned aversion to approach following odor conditioning reinforced by alcohol (Kaun et al., 2011). It is notable that learning-induced plasticity of relative odor-drive to MVP2 persists for at least 3 hr after training whereas output from MVP2 is dispensable for the expression of aversive memory at that time. Since expression of different phases of aversive memory requires distinct combinations of MBON pathways (Bouzaiane et al., 2015), we propose that more persistent MVP2 plasticity might provide a permissive gate for both the formation of aversive memory in, and the expression from, other parts of the MBON network. This would be reminiscent of fear conditioning in the neural circuitry of the mouse amygdala, where dopamine suppresses feed-forward GABA-ergic inhibition from local interneurons to facilitate the induction of long-term potentiation (Bissière et al., 2003).

MVP2 neuron output is required for the expression of sugar-reinforced approach memory at all times. Moreover, odors evoked larger MVP2 responses in hungry than in food-satiated flies, and elevating MVP2 activity in satiated flies promoted inappropriate expression of appetitive memory. These results are consistent with the model that hunger generally increases feed-forward inhibition through MVP2 to support appetitive memory expression (Figure 7). This result is also the mirror-image of that with MP1 DANs whose activity increases when the flies are satiated (Plaçais and Preat, 2013) and whose inhibition leads to the expression of appetitive memory in satiated flies (Krashes et al., 2009). Taken with prior work (Shen and Cai, 2001, Wu et al., 2003, Krashes et al., 2009), we therefore propose that hunger increases dNPF, which releases MP1 inhibition over the KC-MVP2 connection. This results in an increase of odor-evoked MVP2 feed-forward inhibition onto the MBON compartments such as M4/6 that contain the KC-MBON synapses that are directly modified by appetitive conditioning (Figure S6A) (Owald et al., 2015). The increase of MVP2 inhibition into these, and other, compartments allows more efficient expression of the appetitive memory-directed approach behavior by effectively raising the motivational salience of learned food-related odors. Appetitive conditioning may also increase odor-specific recruitment of MVP2 feed-forward inhibition.

Our findings therefore suggest that the MVP2 neuron pathway functions in at least three modes that are presumably selected by the aversively reinforcing MP1 DANs. If the flies are aversively conditioned, phasic MP1 specifically depresses conditioned-odor drive to MVP2 neurons (Figure 7B). In a food-satiated fly, tonic MP1 limits general odor-driven MVP2 activity (Figure 7C). Lastly, in the hungry fly, lower MP1 activity generally enhances odor-drive to MVP2 (Figure 7D). In the OFF modes, low-level MVP2 feed-forward inhibition skews the MBON network toward behavioral avoidance, whereas in the ON mode the increased feed-forward inhibition from MVP2 skews the MBON network toward favoring conditioned approach. The MP1 DANs signal the aversive reinforcing properties of electric shock (Aso et al., 2010, Aso et al., 2012), heat (Galili et al., 2014), and bitter taste (Das et al., 2014), suggesting they provide general aversive influence. The satiated state presumably uses a tonic version of this aversive signal (Plaçais and Preat, 2013) to limit the fly approaching an appetitive odor cue.

The parallels between the fly and mammalian dopaminergic systems appear striking. DANs in the basal ganglia of the mammalian brain also support reinforcement learning and the prediction of stimuli that potentially lead to rewarding outcomes (Bromberg-Martin et al., 2010, Smith et al., 2011, Berridge, 2012, Pignatelli and Bonci, 2015). Furthermore, like the fly DANs, mammalian DANs can be anatomically divided into those that generate aversion and different types of reward (Lammel et al., 2011, Lammel et al., 2012, Cohen et al., 2012, Lerner et al., 2015, Beier et al., 2015, Tellez et al., 2016). GABA-ergic neurons in the mouse ventral tegmental area, whose cell bodies are interspersed with the DANs, have been proposed to signal the value of expected reward and provide a source of subtraction to DANs that calculate a reward prediction error (Eshel et al., 2015). Negatively reinforcing MP1 DANs in the fly modulate odor-drive to the MVP2 neurons to provide the motivational control over actions to gain reward. Therefore, MVP2 neurons may provide an inhibitory bridge between MBON domains that are controlled by aversive and rewarding DANs.

## Experimental Procedures

### Fly Strains

Fly stocks were raised on standard cornmeal food at 25°C and 40%–50% relative humidity. All strain details are provided in the Supplemental Information.

### Behavioral Analysis

Mixed sex populations flies were tested together in all behavior experiments. For the UAS-*Shi*^ts1^ experiments, flies were 4- to 8-day-old and raised at 25°C and 60% relative humidity (activation was 30 min prior to and during the test). For the UAS-*dTrpA1* experiments, the flies were 8 to 11 days old and raised at 20°C and 50% relative humidity (activation was 15 min prior to and during the test). Aversive and appetitive memory were assayed using a T-maze as described previously (Tully and Quinn, 1985, Krashes and Waddell, 2008, Perisse et al., 2013) and as described in more detail in the Supplemental Information.

### Imaging

To visualize native GFP or mRFP, we collected adult flies 4–6 days after eclosion, and brains were dissected in ice-cold 4% paraformaldehyde solution in PBS (1.86 mM NaH_2_PO_4_, 8.41 mM Na_2_HPO_4_, and 175 mM NaCl) and fixed for an additional 60 min at room temperature. Samples were then washed 3 × 10 min with PBS containing 0.1% Triton X-100 (PBT) and 2 × 10 min in PBS before mounting in Vectashield (Vector Labs). Imaging was performed on Leica TCS SP5 X. The resolution of the image stack was 1,024 × 1,024 with 1 μm step size and a frame average of 4. Images were processed in AMIRA 5.3 (Mercury Systems). The immunostaining against GFP, RFP, and GABA was performed as described previously (Burke et al., 2012). We used anti-GFP (chicken, abcam13970, 1:2,000), primary anti-DsRed (Rabbit, Clontech 632496, 1:2,000) and anti-GABA (Rabbit, Sigma A2052, 1:2,000).

### Two Photon Calcium Imaging

3- to 8-day-old UAS-GCaMP6f; MB112C female flies were imaged 30–60 min or 3 to 4 hr after aversive or mock conditioning (Figures 2A–2F) and 30–60 min after appetitive or mock conditioning in a T-maze (Figures S5A and S5B) or following 22–26 hr of starvation or ad libitum feeding (Figure 6A). Flies were trained as described. Imaging experiments were performed essentially as described previously (Owald et al., 2015) and are described in more detail in the Supplemental Information. In brief, flies were anesthetized <10 s on ice and mounted in a custom-made chamber. The head capsule was opened under room temperature sugar-free HL3-like saline (Yoshihara, 2012), and legs and proboscis were immobilized with wax. Fluorescence was excited using ∼140 fs pulses, 80 MHz repetition rate, centered on 910 nm generated by a Ti-Sapphire laser (Chameleon Ultra II, Coherent). Images were acquired using two-photon microscopy (Scientifica) with a 40×, 0.8 NA water-immersion objective, controlled by ScanImage 3.8 software (Pologruto et al., 2003). Odors were delivered on a clean air carrier stream using a custom-designed system (Shang et al., 2007).

### In Vivo Optogenetic Stimulation

Female flies used for optogenetic stimulation were treated as for two photon calcium imaging. Prior to optogenetic experiments all flies were housed on standard cornmeal food with 1 mM retinal for 1–3 days. Saline used was carbogenated (95% O_2_, 5% CO_2_) buffer solution (103 mM NaCl, 3 mM KCl, 5 mM N-Tris, 10 mM trehalose, 10 mM glucose, 7 mM sucrose, 26 mM NaHCO_3_, 1 mM NaH_2_PO_4_, 1.5 mM CaCl_2_, 4 mM MgCl_2_, osmolarity 275 mOsm, pH 7.3). MCH and OCT were presented twice for 2 s, with 20 s inter-trial interval. For light stimulation, a custom-made Labview-triggered LED (Multicomp OSW-6338, 630 nm, 0.85 mW/mm^2^ at specimen) was used at a distance to the brain of 10–15 cm. Light pulses were delivered at 40 Hz, with 10 ms duration, for a total of 1 s per stimulation. The LED was turned on after 1 s of odor onset, during the second round of MCH and OCT presentation. Two-photon fluorescence images were taken from the initial axon segments. A 500/10 filter was used to minimize LED artifacts during imaging. F_0_ was defined as the mean F of the first second of imaging. Time points chosen for comparison were (a) onset of LED, (b) end of LED stimulation, and (c) 1 s after end of LED stimulation.

### Statistical Analysis

Data were analyzed using Matlab and Prism 6 (GraphPad Software). All behavioral data was tested for normality using the D’Agostino and Pearson omnibus test. Normally distributed data were analyzed with one-way ANOVA followed by Tukey’s honest significant difference (HSD) post hoc test. For non-Gaussian distributed data, Kruskall-Wallis test was performed followed by Dunn’s multiple comparison test. Behavioral data from wild-type flies was not included in the statistical analysis. Imaging data were analyzed using Mann-Whitney U-test or two-way repeated-measures ANOVA followed by Sidak’s multiple comparisons test. Definition of statistical significance was set at p < 0.05. Graphs were created in Prism 6.

## Author Contributions

E.P., D.O., and S.W. conceived the project and designed all experiments. E.P. performed and analyzed all behavioral experiments. D.O. and O.B performed imaging experiments with some help from E.P. Imaging data were analyzed by D.O., O.B., and C.B.T. Anatomical data were collected by E.P., D.O., and W.H. The manuscript was written by S.W., E.P., and D.O.

## Supplemental Information

Document S1. Figures S1–S6, Movies S1–S3 and Supplemental Experimental Procedures

Movie S1. Projection View of the MVP2 Neurons Projection of MB112C driven UAS-mCitrine reveals the detailed 3D morphology of MVP2 processes.

Movie S2. Projection View of the Innervation of the MVP2 and M4/6 Neurons in the Horizontal Lobe Tip of the MB Genotype; R83A12-GAL4; UAS-GCaMP6f (MVP2, orange) and R21D02-LexA; lexAop-CD2::mRFP (M4/6 cyan).

Movie S3. Projection View of the Innervation of the MVP2 and V2αV2α′ Neurons in the Vertical Lobe of the MB Genotype; R12G04-LexA; lexAop-rCD2::mRFP (MVP2, orange) and R71D08-GAL4; UAS-mCD8::GFP (V2αV2α′, cyan).

Document S2. Article plus Supplemental Information
