# Supplementary material for: Aversive Learning and Appetitive Motivation Toggle Feed-Forward Inhibition in the Drosophila Mushroom Body
Source: Neuron. 2016 Jun 1;90(5):1086–99. doi: 10.1016/j.neuron.2016.04.034 (PMC4893166; doi:10.1016/j.neuron.2016.04.034)
Supplement: Document S1. Figures S1–S6, Movies S1–S3 and Supplemental Experimental Procedures [file mmc1.pdf]

Neuron, Volume *90*

Supplemental Information

Aversive Learning and Appetitive Motivation Toggle Feed-Forward Inhibition in the *Drosophila* Mushroom Body

Emmanuel Perisse, David Owald, Oliver Barnstedt, Clifford B. Talbot, Wolf Huetteroth, and Scott Waddell

Fly Strains

The wild-type *Drosophila* strain used in this study is Canton-S. UAS-mCD8::GFP (Lee and Luo, 1999), 247-LexA::VP16 (Pitman et al., 2011), lexAop-rCD2::RFP (Lai and Lee, 2006), UAS-DenMark (Nicolai et al., 2010) and UAS-GFP-Syd-1 (Owald et al., 2010) flies are described. The MVP2 neuron (MBON-γ1pedc>αβ) expressing lines, R83A12-GAL4, MB112C-GAL4 and R12G04-LexA , as well as the M4/6 expressing line R21D02-GAL4 (described in Owald et al., 2015) and the V2αV2α´expressing line R71D08-GAL4 are from the FlyLight collection (Jenett et al., 2012; Aso et al., 2014b). UAS-GCaMP6f is that in Chen et al. (2013); these strains were obtained from the Bloomington Stock Center. The lexAop-CsChrimson-tdtomato,UAS-GCaMP6f flies are described (Hoopfer et al., 2015). The MP1 (PPL1-γ1pedc) DAN expressing c061:MBGAL80 combination is that in Krashes et al. (2009). We used flies carrying UAS-shi^{ts1} (Kitamoto, 2001) on the third chromosome. UAS-*dTrpA1* flies are described (Hamada et al., 2008). We generated flies expressing shi^{ts1} or *dTrpA1* in MVP2 drivers by crossing UAS-shi^{ts1} or UAS-*dTrpA1* females to R83A12 and MB112C males. For imaging MB112C and UAS-GCaMP6f were combined. We generated flies expressing shi^{ts1} or *dTrpA1* in MP1 neurons by crossing c061:MBGAL80 females to UAS-shi^{ts1} or UAS-*dTrpA1* males. For the optogenetic experiments we used the appropriate female progeny from crosses between lexAop-CsChrimson-tdtomato,uas-GCaMP6f males and R12G04-LexA/CyO;R21D02-GAL4 or R12G04-LexA/CyO;R71D08-GAL4/TM3 females.

Behavioral analysis

For appetitive memory groups of ~100 flies were food-deprived in a 25 ml vial, containing 1% agar and a 20 x 60 mm piece of filter paper for 19–23 h before training. Flies were conditioned as follows: 2 min with odor A without reinforcement, 30 s of air, 2 min with odor B with saturated ~5.8M sucrose, dried on a filter paper. To test 30 min, 3 h or 24 h memory flies were trained and stored in food vials, or food-deprivation vials, until testing.

For aversive memory, groups of ~100 flies were housed for 18–24 h before training in a 25 ml vial containing standard cornmeal/agar food and a 20 x 60 mm piece of filter paper. Flies were conditioned as follows: 1 min odor A with 12 120 V shocks at 5 s interstimulus interval, 45 s air, and 1 min odor B without reinforcement.

Memory performance was tested in the dark by allowing the flies 2 min to choose between the odors presented during training. Performance index (PI) was calculated as the number of flies approaching (appetitive memory) or avoiding (aversive memory) the conditioned odor minus the number of flies going the other direction, divided by the total number of flies in the experiment. A single PI value is the average score from flies of the identical genotype tested with the reciprocal reinforced/ non-reinforced odor combination. The odors used for conditioning were 3-octanol (7 µl in 8 ml mineral oil) and 4-methylcyclohexanol (7 µl in 8 ml mineral oil).

Naïve avoidance was performed as described (Owald et al., 2015). Fed flies were transferred to 33 °C 30 min (for the *shi*^{ts1} experiments) or 15 min (for the *dTrpA1* experiments) before a 2 min test in the dark between MCH or OCT diluted in mineral oil (1:10^{-6}) versus mineral oil.

Two photon calcium imaging

One hemisphere per fly was randomly chosen and imaged. Two-photon fluorescence images were manually segmented using ImageJ. Animal movement was small enough that images did not require registration. Fluorescence over the defined region of interest (ROI) was summed at each frame to yield one fluorescence trace, F(t). The protocol to test responses after training was that in Owald et al. (2015). Flies were exposed to 5 s MCH (air stream passing over 10^{-2} odor dilution in mineral oil, and then further blended 1:9 with a clean air stream), 30 s clean air, followed by 5 s OCT. This odor regimen was delivered twice. Baseline fluorescence (F) corresponds to the average fluorescence signal across an 8 s window starting 9 s after scan onset and terminating 3 s before the first air or odor exposure. Baseline was then used to compute the relative change in fluorescence (ΔF(t)/F = (F(t) – F)/F). Responses were determined to start approximately 2.25 s after the instrumentation odor delivery command and to end within 12.5 s. This delayed onset accounts for the computational, electronic, mechanical and fluid flow lag. We corrected for small changes in the background fluorescence between odor presentations by subtracting the mean fluorescence between 0 and 2.25 s from each curve. The response curves were normalized and averaged over the two paired odor presentations:

$$\mathrm{CS}_n^{+/-}(t) = \frac{1}{2}\sum_{i=1}^{2} \frac{\mathrm{CS}_{n,i}^{+/-}(t)}{\int_0^{12.5}\left(\frac{\mathrm{CS}_{n,i}^{+}(t) + \mathrm{CS}_{n,i}^{-}(t)}{2}\right)dt}$$

Equation (i)

$\mathrm{CS}_{n,i}^{+/-}(t)$ are the background subtracted ΔF(t)/F response curves of the n'th experiment to the i'th odor stimulation protocol. The normalization factor was chosen to be the average of the total CS+ and CS- response to avoid bias towards one or the other and was calculated as the sum over the acquisition time points of the ΔF(t)/F curves multiplied by the sampling interval. We (1) computed the mean responses for all normalized CS+ and CS- responses and (2) computed the odor response difference for each n, $D_n(t) = CS_n^+ - CS_n^-$. To quantify the difference between the trained and mock groups, the area under the peak of each D_n curve (defined as 4.5± 1.5 s after odor delivery for experiments following training) was computed following Owald et al. (2015).

The peak values obtained from each trained group were compared with those of the corresponding mock group using the Mann-Whitney U-test. The learning-induced difference curve, L(t), is the difference between the mean ± SEM of the $D_n(t)$ curves of the trained and corresponding mock groups. The errors were combined in the usual way – i.e. error in $\mathrm{L}(t) = \sqrt{\mathrm{SEM}(t)_{trained}^2 + \mathrm{SEM}(t)_{mock}^2}$. For naïve odor response experiments (Fig. 6A) only one round of odors was applied per fly; the intervals between odor presentation onset was 30 s. Odor response traces were averaged across flies per group and thus no normalization was applied. Peaks of naïve traces were also estimated at 4.5± 1.5 s across odors.

Supplementary References

Lai, S. L., and Lee, T. (2006). Genetic mosaic with dual binary transcriptional systems in Drosophila. Nat. Neurosci. *9*, 703-709.

Lee, T., and Luo, L. (1999). Mosaic analysis with a repressible cell marker for studies of gene function in neuronal morphogenesis. Neuron *22*, 451-461.

Pitman, J. L., Huetteroth, W., Burke, C. J., Krashes, M. J., Lai, S. L., Lee, T., and Waddell, S. (2011). A pair of inhibitory neurons are required to sustain labile memory in the Drosophila mushroom body. Curr. Biol. *21*, 855-861.

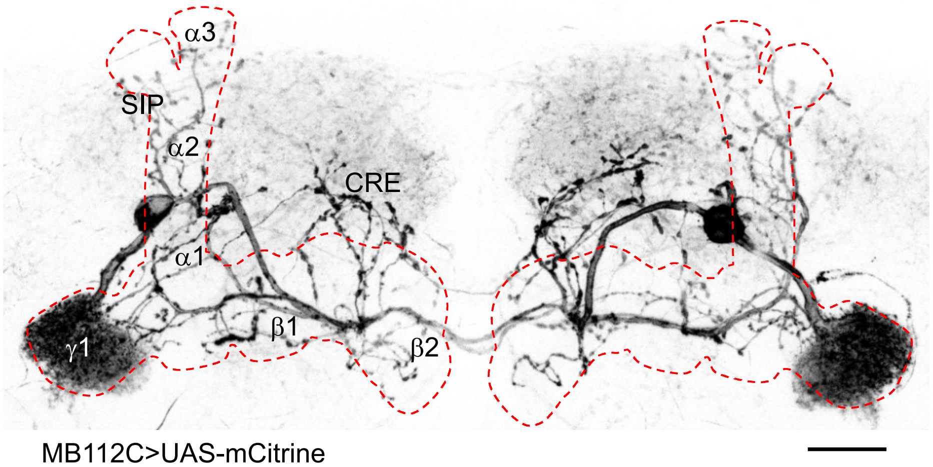

MB112C>UAS-mCitrine

Figure S1. Anatomy of MVP2 neuron projections within and outside the MB (related to Figure 1).

Black and white confocal projection of MB112C driven UAS-mCitrine reveals the detailed morphology of MVP2 processes. The dendritic field lies in the $\alpha\beta_s$ a□□ γ1 zone whereas presynaptic processes innervate the α1, α2, α3, β1 and β2 compartments of the MB lobes as well as outside the MB in the crepine (CRE) and superior intermediate protocerebrum (SIP).

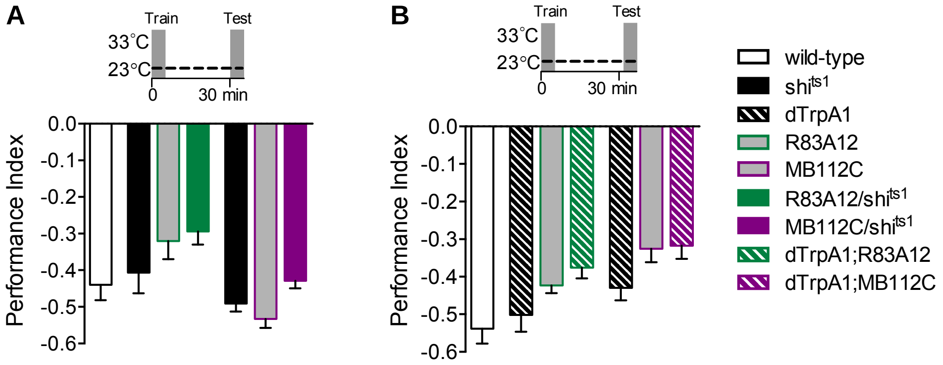

Figure S2. Expressing UAS-*shi*^{ts1} or UAS-*dTrpA1* in MVP2 neurons does not disrupt 30 min aversive memory performance at the permissive temperature (related to Figure 2).

(A) No statistical differences were apparent between MVP2 expressing UAS-*shi*^{ts1} flies and their relevant GAL4 or UAS-*shi*^{ts1} controls (R83A12: ANOVA, n = 8, P > 0.2. MB112C: ANOVA, n = 10-12, P < 0.01. (B) No statistical differences in 30 min aversive memory performance were apparent between MVP2 expressing UAS-*dTrpA1* flies and their relevant GAL4 or UAS-*dTrpA1* controls (R83A12: ANOVA, n = 8-10, P = 0.03. MB112C: ANOVA, n = 8-10, P > 0.05). All flies were trained, stored and tested at 23 °C. All data are mean ± SEM.

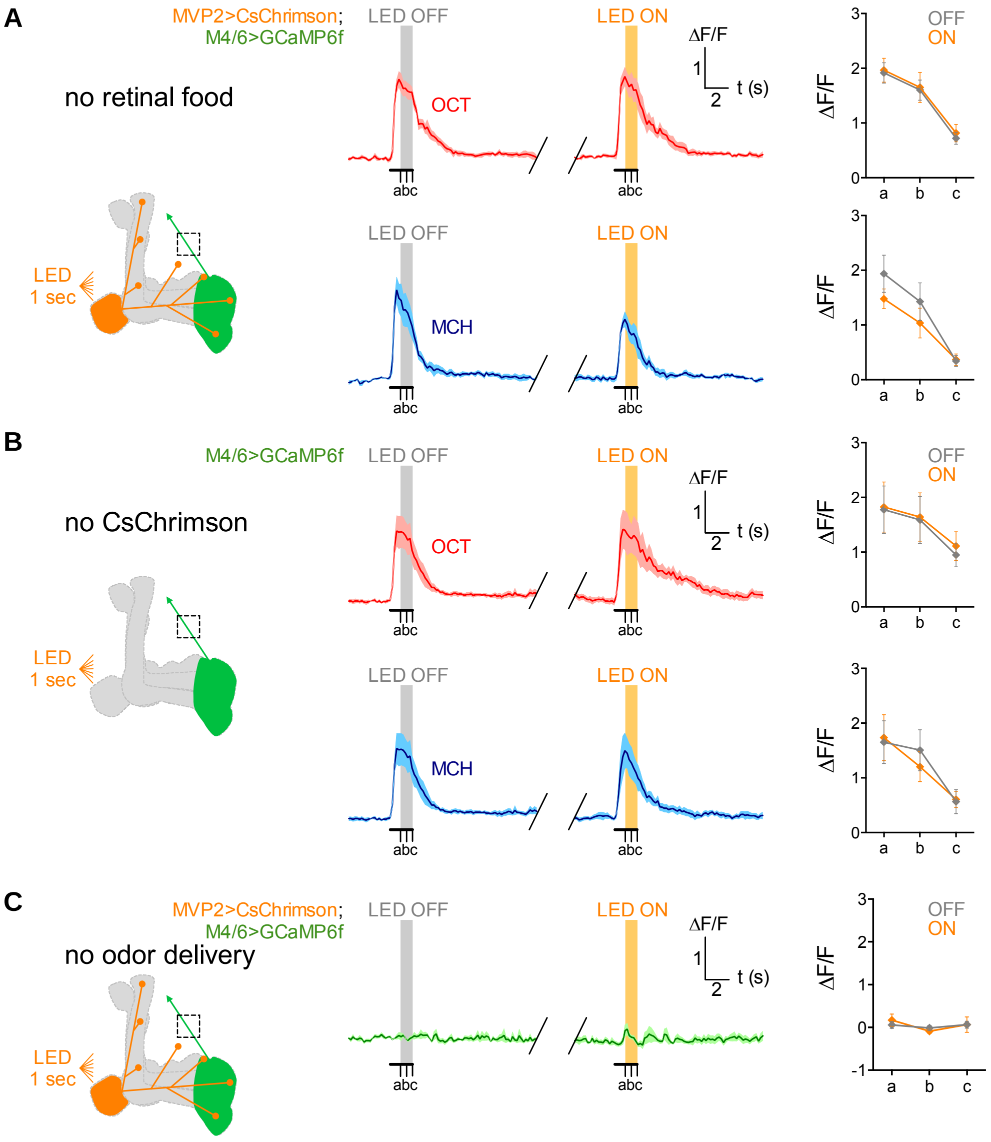

Figure S3. Control experiments related to Figure 4

Odor evoked GCaMP6f responses were measured in M4/6 MBON axons (green). Left panels, schematics of experiment. (A) Flies not fed with retinal before the experiment. Calcium traces during OCT (top middle panel, red) or MCH (bottom middle panel, blue) presentation show robust odor-evoked responses in M4β´ axons. Data are mean [solid line] ± SEM [shaded area]. LED ON illumination for one second did not change the odor-evoked responses compared to the LED OFF condition. Quantification of the ΔF/F at the a-c time points did not reveal a significant difference in the odor-evoked responses with LED ON (orange) compared to the same time point with LED OFF (grey), for either OCT (top right) or MCH (bottom right) (Both two-way repeated measures ANOVA, no interaction effect $P > 0.2$, n =

5). (B) Flies lacked the UAS-CsChrimson transgene. Calcium traces during OCT (middle top panel, red) or MCH (middle bottom panel, blue) presentation show robust odor-evoked responses in M4β´ axons. Data are mean [solid line] ± SEM [shaded area]. LED ON did not impair odor-evoked calcium responses compared to the LED OFF condition. Quantification of the ΔF/F at the a-c time points did not reveal a significant difference between the conditions; LED ON (orange), LED OFF (grey), for either OCT (top right) or MCH (bottom right) (Both two-way repeated measures ANOVA, no interaction effect $P > 0.05$, $n = 5$). (C) Flies were not exposed to odors. Data are mean [solid line] ± SEM [shaded area]. Triggering MVP2 activity with LED ON for one second did not reveal an obvious change in calcium signal in M4β´ axons compared to the LED OFF condition. Quantification of the ΔF/F at the a-c time points did not reveal a significant difference between the conditions; LED ON (orange), LED OFF (grey) (Two-way repeated measures ANOVA, no interaction effect $P > 0.4$, $n = 5$).

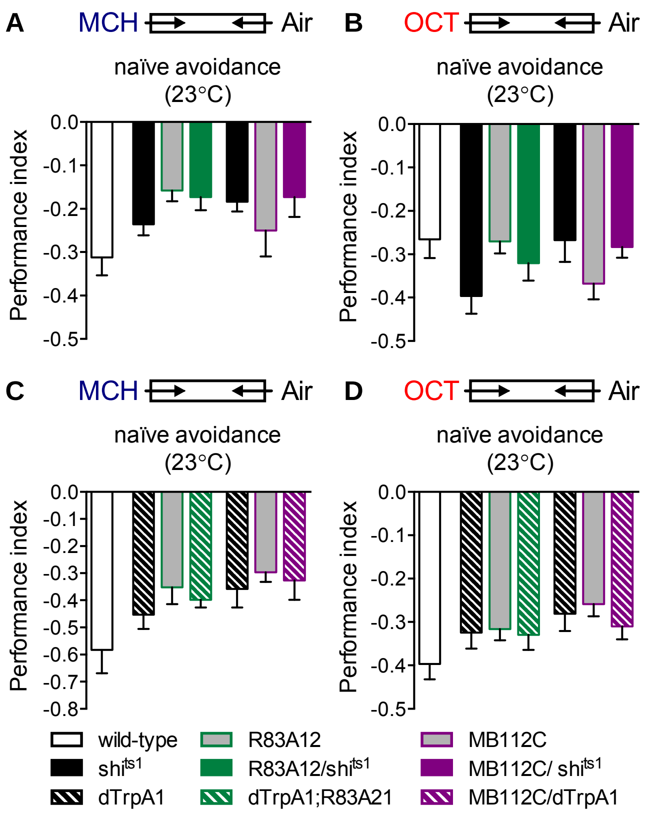

Figure S4. UAS-*shi*^{ts1} or UAS-*dTrpA1* expression in MVP2 neurons does not alter odor avoidance behavior at permissive temperature (related to Figure 5)

Flies chose between T-maze arms perfused with MCH or OCT versus a clean air stream at 23°C. (A-B) No significant differences in naïve odor avoidance were apparent between MVP2;UAS-*shi*^{ts1} flies and their relevant GAL4 or UAS-*shi*^{ts1} controls for (A) MCH (R83A12: ANOVA, n = 10-15, P > 0.1. MB112C: ANOVA, n = 8-13, P > 0.4) or for (B) OCT (R83A12: ANOVA, n ≥ 16-20, P < 0.05. MB112C: ANOVA, n = 10-17, P > 0.01). (C-D) No statistical differences in naïve odor avoidance were apparent between MVP2; UAS-*dTrpA1* flies and their relevant GAL4 or UAS-*dTrpA1* controls for (C) MCH (R83A12: ANOVA, n = 10-15, P > 0.3. MB112C: ANOVA, n = 10-12, P > 0.7) or for (D) OCT (R83A12: ANOVA, n = 11-17, P > 0.9. MB112C: ANOVA, n = 12, P > 0.5).

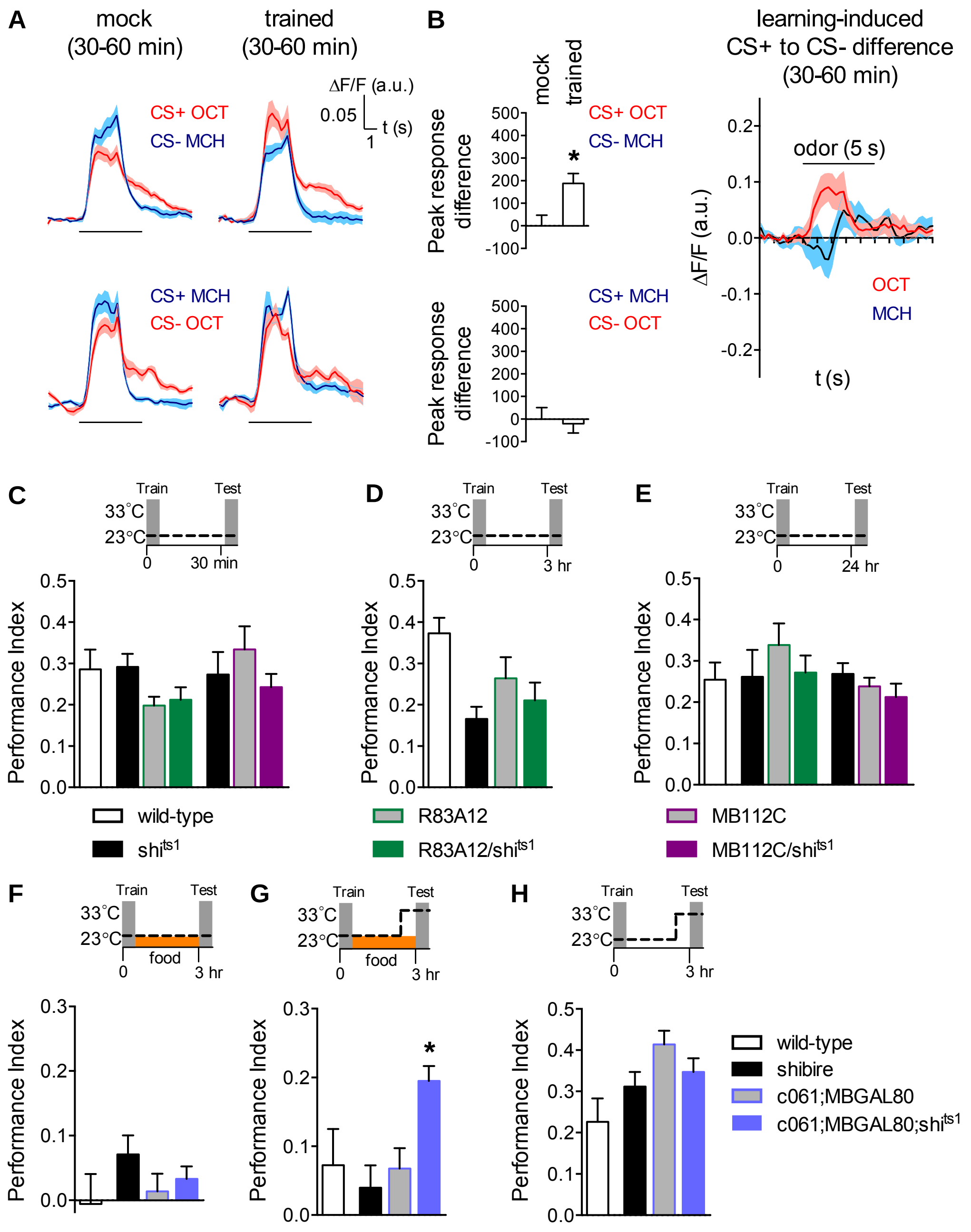

Figure S5. Control experiments related to Figure 6

(A) Flies were trained by pairing one odor with sugar (CS+) and another odor without (CS-), or they were mock trained with only odor exposure. Odor-evoked calcium transients were measured 30-60 min after conditioning. Appetitive conditioning; CS+ (normalized mean ± SEM) to CS- (normalized mean ± SEM). (B) Bar graphs represent percent difference to the mean mock integrated peak response (4.5 ± 1.5s after odor delivery, see methods). Asterisk indicates statistical significance. (Mann-Whitney U-test; OCT is CS+: n(mock) = 9, n(trained) = 11, $P < 0.05$. MCH is CS+:

n(mock) = 7, n(trained) = 7, P > 0.05; curves are normalized, see methods). Difference of responses evoked by CS+ and CS- after appetitive conditioning relative to the mean responses after mock training (red curve: OCT is CS+, blue curve: MCH is CS+). Data are mean [solid line] ± SEM [shaded area]. (C-E) All flies were trained with odors and sugar reward and tested for memory performance at 23°C. No statistical differences were apparent between any groups and their relevant controls. (C) 30 min (R83A12: ANOVA, n = 8-10, P > 0.05. MB112C: ANOVA, n = 8, P > 0.4). (D) 3 h (R83A12: ANOVA, n = 10-11, P > 0.2). (E) 24 h (R83A12: ANOVA, n = 8-10, P > 0.5. MB112C: ANOVA, n = 12-14, P >0.3). (F) Feeding flies after training suppresses appetitive memory performance. Flies were trained at 23 °C, stored in food vials and tested for 3 h memory performance at 23 °C. No statistical differences were apparent between flies expressing UAS-shi^{ts1} in MP1 neurons (c061;MBGAL80) and their relevant controls (ANOVA, n = 10, P > 0.2). (G) Blocking MP1 DANs 30 min prior to and during testing promotes the expression of appetitive memory (ANOVA, n = 10-12, P < 0.001). Flies were trained, stored in food vials and tested for 3 h memory at 33°C. (H) Blocking MP1 DANs in hungry flies did not further elevate 3 h appetitive memory performance (ANOVA, n = 8, P > 0.1). Data are mean ± SEM.

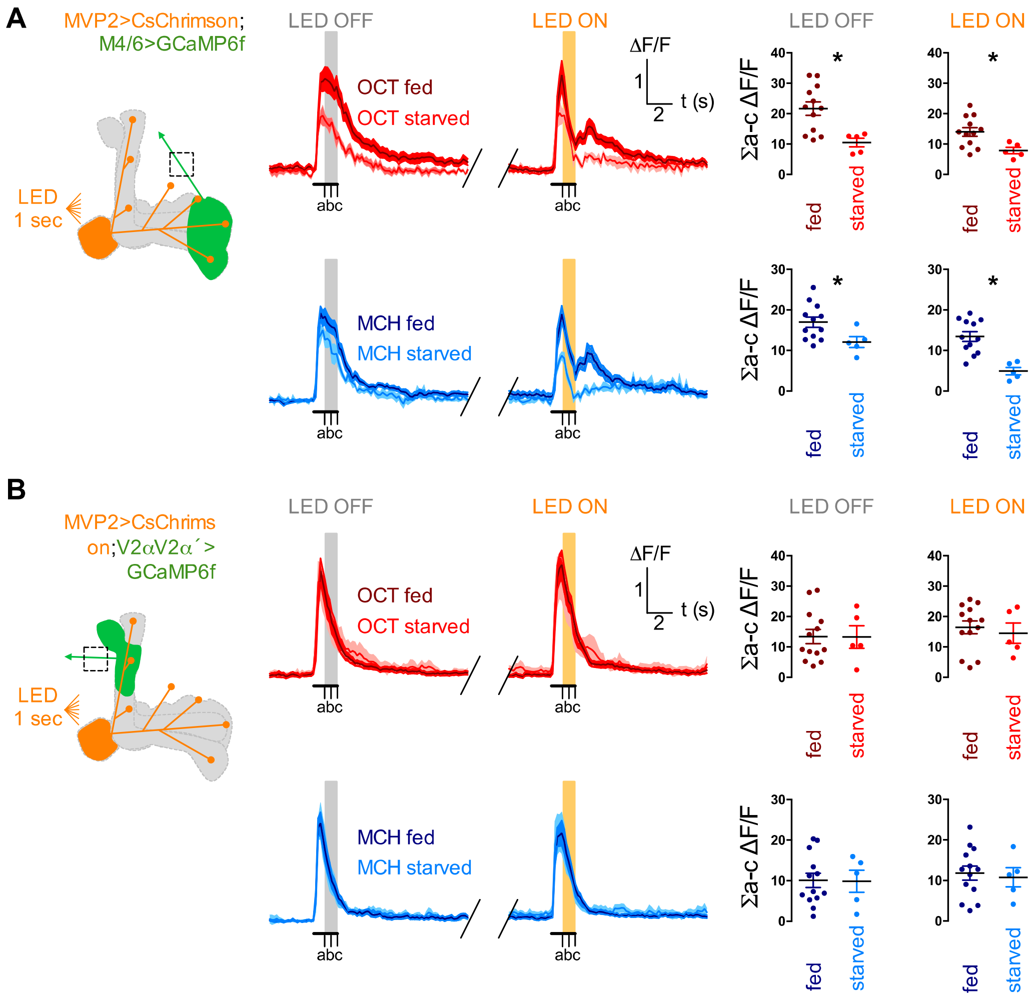

Figure S6. Odor-evoked activity in M4/6 but not V2αV2α´ MBONs is reduced in hungry flies (related to Figure 4 and 6).

Left panels, schematics of experiment. Odor evoked GCaMP6f responses were measured in (A) M4/6 or (B) V2αV2α´ MBON axons (black dashed box on green arrow) while CsChrimson-expressing MVP2 MBONs (orange) were light-triggered. Responses compared between fed (data from Figure 4) and starved flies. (A) OCT (middle top panel, red) and MCH (middle bottom panel, blue) evoked calcium transients in M4β´ axons are markedly smaller in starved versus fed flies. Activating MVP2 neurons with LED ON for one second caused a clear depression in the calcium transient in both conditions. Data are mean curves [solid line] ± SEM [shaded area]. Quantifying the area under the curve of the ΔF/F during the odor presentation (∑a-c) shows a significant difference in the starved responses compared to fed for both OCT (top right panels) and MCH (bottom right panels) and between LED OFF and LED ON. (All Mann-Whitney, $P < 0.05$, $n = 5\text{-}12$). (B) OCT

(middle top panel, red) and MCH (middle bottom panel, blue) evoke similar calcium responses in V2αV2α´ axons in starved and fed flies. Triggering MVP2 neurons with LED ON (orange) for one second did not alter the calcium responses in either condition. Data are mean curves [solid line] ± SEM [shaded area]. Quantifying the area under the curve of the ΔF/F during the odor presentation (∑a-c) did not reveal any statistical differences in V2αV2α´ OCT (right top panels) or MCH (right bottom panels) odor responses between fed or starved flies, or for LED OFF versus LED ON. (All Mann-Whitney, $P > 0.7$, $n = 5\text{-}13$).

Movie S1, related to Figure 1 and S1. Projection view of the MVP2 neurons. Projection of MB112C driven UAS-mCitrine reveals the detailed three-dimensional morphology of MVP2 processes.

Movie S2, related to Figure 3 and 4. Projection view of the innervation of the MVP2 and M4/6 neurons in the horizontal lobe tip of the MB. R83A12-GAL4; UAS-GCaMP6f (MVP2, orange) and R21D02-LexA; lexAop-rCD2::mRFP (M4/6 cyan).

Movie S3, related to Figure 3 and 4. Projection view of the innervation of the MVP2 and V2αV2α´ neurons in the vertical lobe of the MB. R12G04-LexA; lexAop-rCD2::mRFP (MVP2, orange) and R71D08-GAL4; UAS-mCD8::GFP (V2αV2α´, cyan).
