# Supplementary figures and images for: Aversive Learning and Appetitive Motivation Toggle Feed-Forward Inhibition in the Drosophila Mushroom Body
Source: Neuron. 2016 Jun 1;90(5):1086–99. doi: 10.1016/j.neuron.2016.04.034 (PMC4893166; doi:10.1016/j.neuron.2016.04.034)

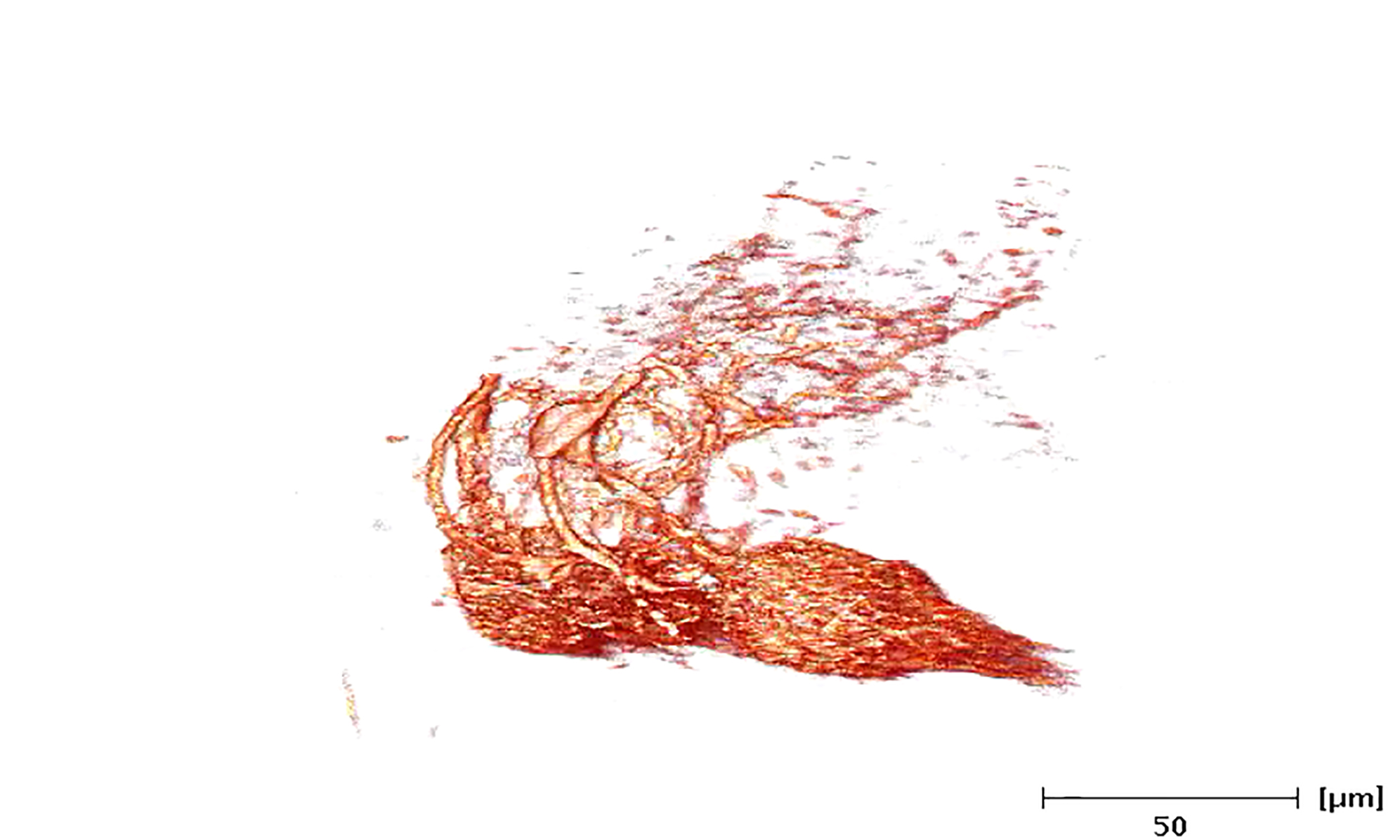

Supplement: Movie S1. Projection View of the MVP2 Neurons — Projection of MB112C driven UAS-mCitrine reveals the detailed 3D morphology of MVP2 processes. [file mmc2.jpg]

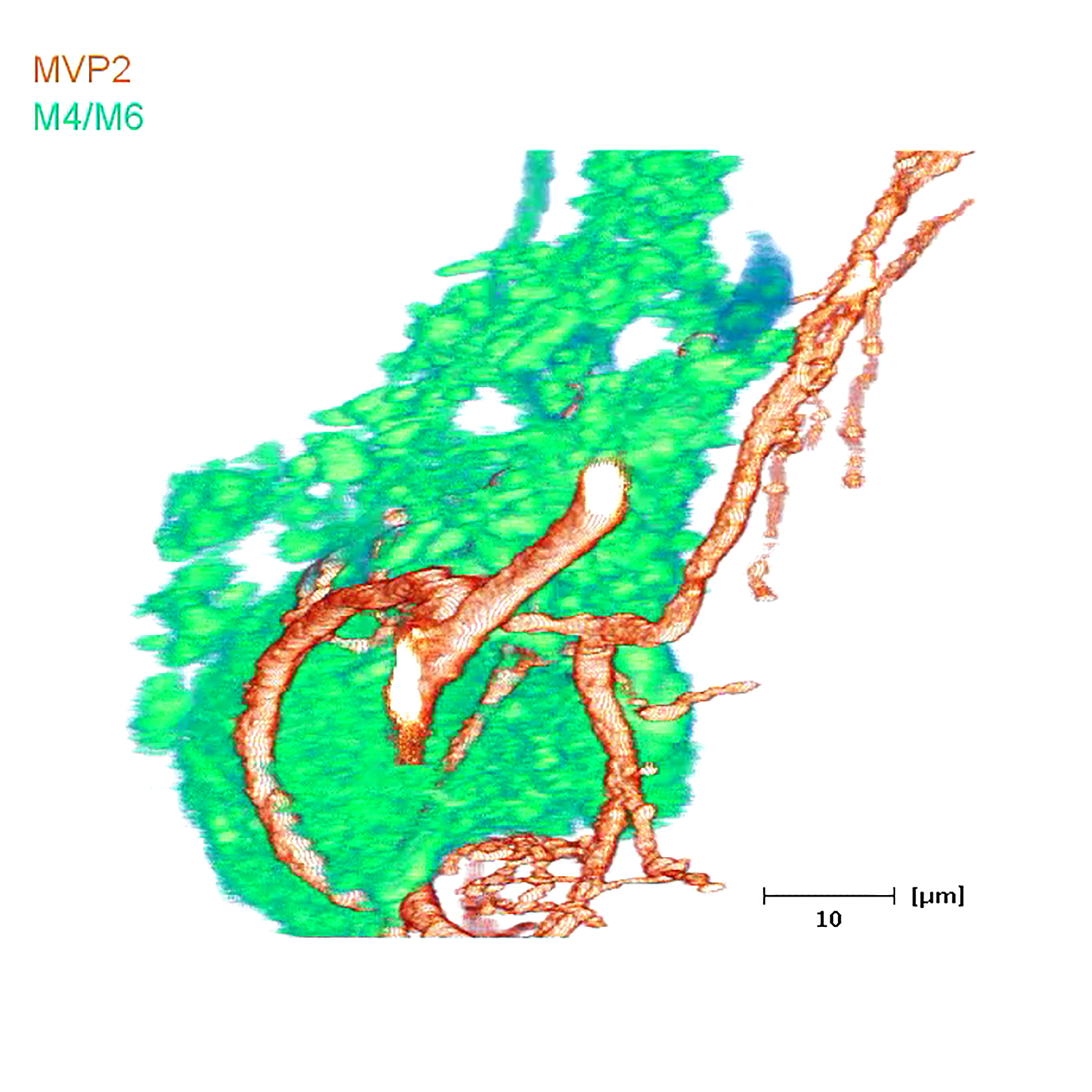

Supplement: Movie S2. Projection View of the Innervation of the MVP2 and M4/6 Neurons in the Horizontal Lobe Tip of the MB — Genotype; R83A12-GAL4; UAS-GCaMP6f (MVP2, orange) and R21D02-LexA; lexAop-CD2::mRFP (M4/6 cyan). [file mmc3.jpg]

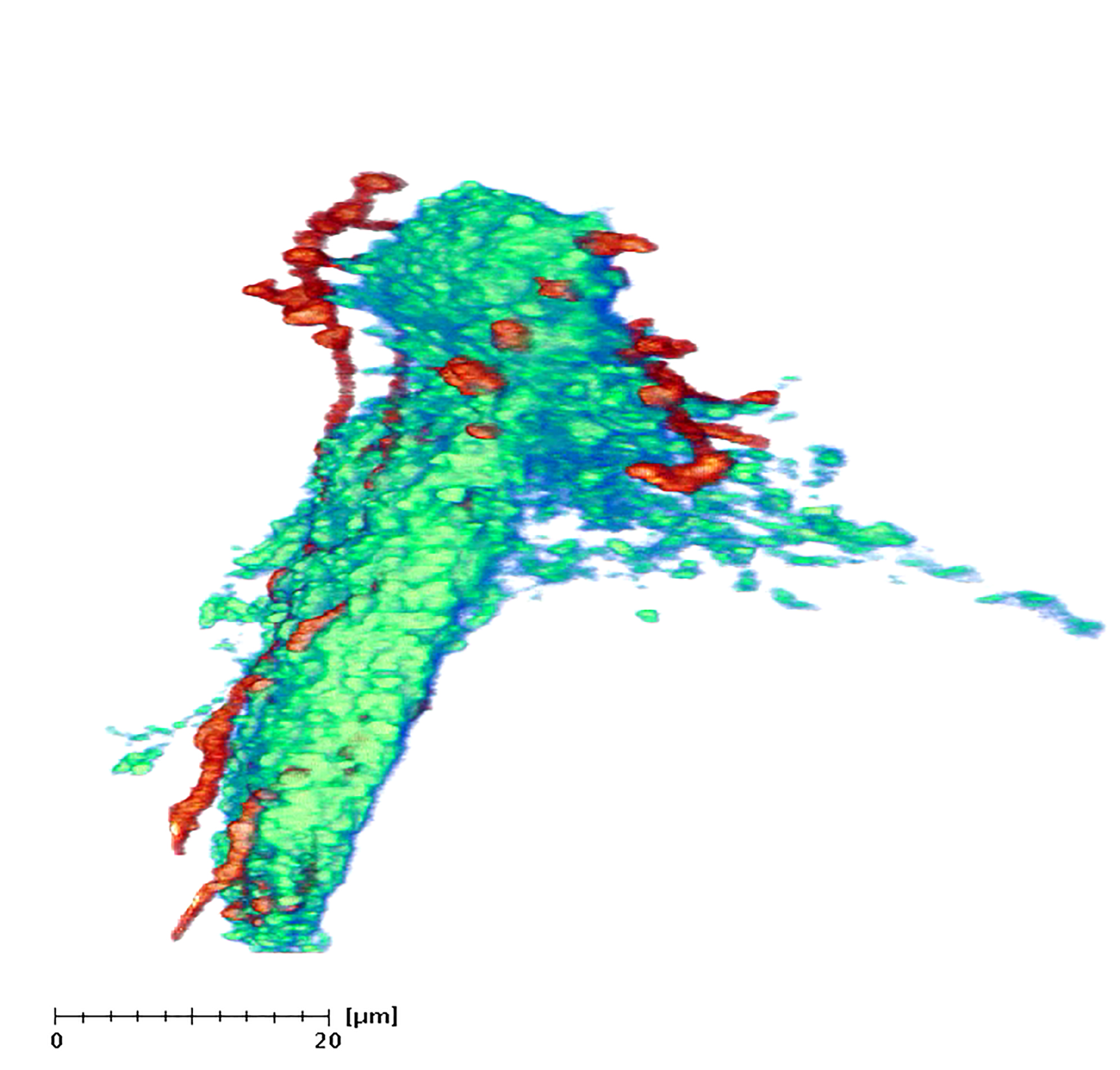

Supplement: Movie S3. Projection View of the Innervation of the MVP2 and V2αV2α′ Neurons in the Vertical Lobe of the MB — Genotype; R12G04-LexA; lexAop-rCD2::mRFP (MVP2, orange) and R71D08-GAL4; UAS-mCD8::GFP (V2αV2α′, cyan). [file mmc4.jpg]
